# Magnetic Antiaromaticity—Paratropicity—Does Not Necessarily Imply Instability

**DOI:** 10.1021/acs.joc.3c01807

**Published:** 2023-09-29

**Authors:** Cina Foroutan-Nejad

**Affiliations:** Institute of Organic Chemistry, Polish Academy of Sciences, Kasprzaka 44/52, 01-224 Warsaw, Poland

## Abstract

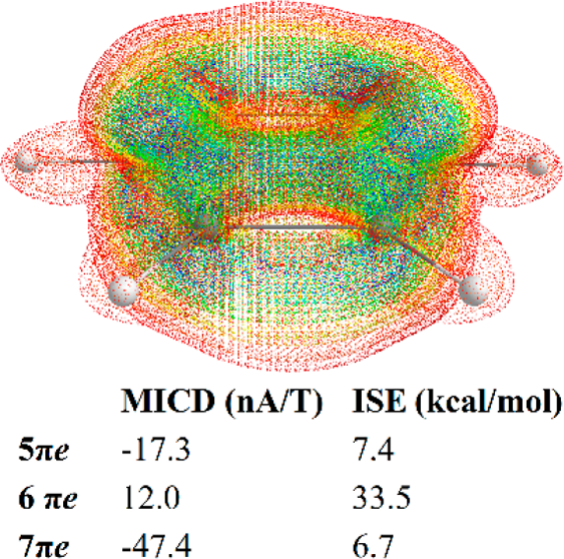

Magnetically induced ring currents are a conventional tool for the characterization of aromaticity. Dia- and paratropic currents are thought to be associated with stabilization (aromaticity) and destabilization (antiaromaticity), respectively. In the present work, I have questioned the validity of the paratropic currents as a measure of antiaromaticity among monocyclic hydrocarbons. I have shown that while reduced/oxidized radical ions of hydrocarbons sustain strong paratropic currents, they often gain extra stabilization via cyclic conjugation compared to their acyclic counterparts.

The concept of (anti)aromaticity has been one of the most employed tools to explain reactivity and stability within the context of chemistry.^1^ This concept not only proved to be useful for hydrocarbons in their electronic ground states but also has been vastly used to interpret characteristics of metallaaromatics^2,3^ and metallic clusters^4−6^ as well as hydrocarbons in their excited electronic states.^7,8^ However, because aromaticity does not have a quantum mechanical operator, qualitative and quantitative assessment of aromaticity proved to be a controversial task. Aromaticity criteria, i.e., energetic,^9^ structural,^10^ electronic,^11^ and magnetic,^12,13^ sometimes conflict with one another.^14−17^ Even within a particular criterion of aromaticity, sometimes different tools show contradictory results or at least results that are open for discussion.^18−23^ Even the validity of the Hückel rule and the role of active orbitals in the magnetic response of the aromatic species has been questioned recently.^14^ In a recent perspective article, my colleagues and I collected opinions of several experts in the field of aromaticity, hoping to reach an agreement on the topic.^1^ However, different responses to similar questions proved that we have a long way to reach a consensus on the concept of aromaticity.^24^ In the meantime, we may gather more information in favor or against some ideas to prove or rule out some theories of aromaticity.

Here, I study the magnetic and energetic properties of radical ions of seven monocyclic hydrocarbons, which are all well-known 6π-electron species, Figure 1. Among these species, the radical cation and anion of benzene have been extensively studied in the past decades based on various criteria of aromaticity.^25−30^ Interestingly, these studies often come to different conclusions on the aromatic nature of benzene’s radical ions. While Dietz et al. classified both the radical cation and the radical anion of benzene as antiaromatic,^28^ Mandado et al. defined the benzene radical cation as antiaromatic but considered the corresponding radical anion as aromatic.^29^ Mandado and his colleagues further proposed an updated electron-counting rule for the definition of (anti)aromaticity. According to their rule, any species with an odd number of α/β electrons is aromatic. For instance, benzene with 3α-π and 3β-π electrons is double α and β aromatic. However, the aromaticity of benzene’s radical ions is the result of conflicting α and β aromaticity. Ottosson and co-workers verified this suggestion in a recent preprint on singly excited monocyclic aromatic rings.^31^ Later, Andjelković et al. classified both the radical anion and the radical cations of benzene as nonaromatic.^30^ Indeed, the tools and the chosen theoretical/experimental methods have a significant effect on the conclusions drawn regarding the benzene’s radical ions.

**Figure 1:**
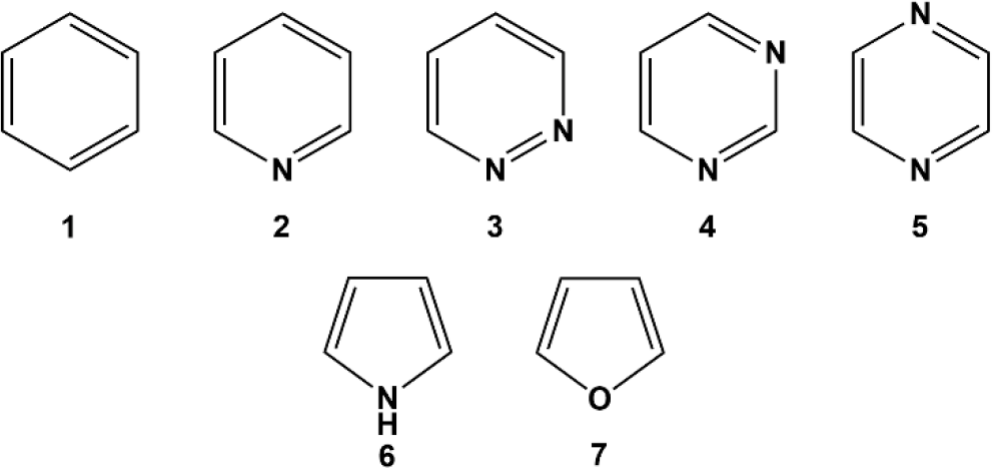
Structures of the studied molecules: (**1**) benzene, (**2**) pyridine, (**3**) 1,2-diazine, (**4**) 1,3-diazine, (**5**) 1,4-diazine, (**6**) pyrrole, and (**7**) furan.

In the following, I show evidence that paratropic currents, often associated with antiaromaticity, do not necessarily imply energetic instability. Although it is known that stable metallic clusters^15,19^ and porphyrinoids^16^ might sustain paratropic currents, to the best of my knowledge, stable monocyclic hydrocarbons and heterocycles with strong paratropic currents have not been reported so far.

The structures of radical ions of 7 molecules were optimized in their electronic ground states. The plots of spin density, Figure S2, besides MO analysis, suggest that all radical anions except for the radical anion of pyrrole accommodate the extra electron in their π-MOs and form a 7π-electron system. The radical anion of pyrrole has a 2A′ electronic state, and the extra electron mainly occupies σ* of the N–H bond to form a 2*c*3*e* bond based on the MO morphology and spin density analysis, Figure S2.^32^ Among the radical cations at their electronic ground state, the electron is removed from the lone pairs of the heteroatoms in pyridine and all diazines (**2**–**5**); just three molecules, benzene, pyrrole, and furan, have radical cations with 5π electrons at the selected computational level (B3LYP/def2-TZVPP). The 6π-electron radical cations of pyridine and diazines sustain diatropic currents that are comparable to those of their neutral parents. It is worth noting that in a study that was published while I was preparing this article, Wu and her co-workers identified that the M11^33^ functional works the best for antiaromatic systems.^34^ I repeated the computations at the M11/def2TZVPP level and found the same trend as the one reported here. Further information is given in Methods in the SI.

The current intensity of all radical ions, except for those of furan, is dominated by paratropic currents that are conventionally considered to be a sign of antiaromaticity within the context of the magnetic criterion of aromaticity. The paratropic currents of radical ions are comparable with those of known antiaromatic cyclobutadiene (MICD = −20.0 nA/T at the B3LYP/def2-TZVPP level and −15.9 nA/T at the CAS(12,12)/aug-cc-pVDZ level as reported by Ruud and his co-workers).^35^ The change of the ring current from diatropic to paratropic can be understood based on the selection rules introduced by Steiner and Fowler.^36^ According to Steiner and Fowler’s rules, the nature of the ring current is determined by the virtual transitions between the occupied and the unoccupied orbitals around the Fermi level of a molecule, i.e., HOMOs and LUMOs. To simplify the rules, one can state that any transition between orbitals having the same nodal symmetry results in paratropic currents. A transition between orbitals with different nodal symmetries contributes to the diatropic currents. The intensity of the current is determined by the spatial distribution and the energy difference between the occupied and the unoccupied orbitals involved in the process. In 6π-aromatic systems, the number of nodal planes perpendicular to the ring plane in the π MOs increases from 0 to 1, 2, and 3. Transitions between π-MOs with the same number of nodal planes result in paratropicity, but transitions from an MO with a lower number of nodal planes to one with a higher number of nodal planes result in diatropicity. In benzene, for example, the main transition is from e_1g_ (degenerate HOMO with one nodal plane perpendicular to the ring plane) to e_2u_ (degenerate LUMOs with two nodal planes perpendicular to the ring plane) of the molecule that causes a dominant diatropic current, Figure 2. In radical cations, an electron is removed from the HOMO, and the degeneracy between HOMOs is disturbed through the Jahn–Teller distortion. Therefore, the electron transition between the HOMO with one perpendicular plane of symmetry to the ring plane and the SOMO with the same symmetry and just slightly higher energy is now permitted. This transition should contribute to a strong paratropic ring current. In radical anions, the SOMO of the molecules has two perpendicular nodal planes to the ring plane of the molecule. The transition between the SOMO and a low-energy LUMO with the same symmetry can induce a substantial paratropic current, again. To test this hypothesis, I computed the current intensity of benzene radicals at the BLYP/def2-TZVPP level. Pure GGA methods are known to reduce the Fermi gap of the molecule, which is expected to increase the current intensity. The MICD values obtained from the BLYP computations are larger than those of B3LYP, which is consistent with the expected change (MICD_BLYP_ = −55.3 and −27.0 nA/T for the benzene radical anion and cation, respectively).

**Figure 2:**
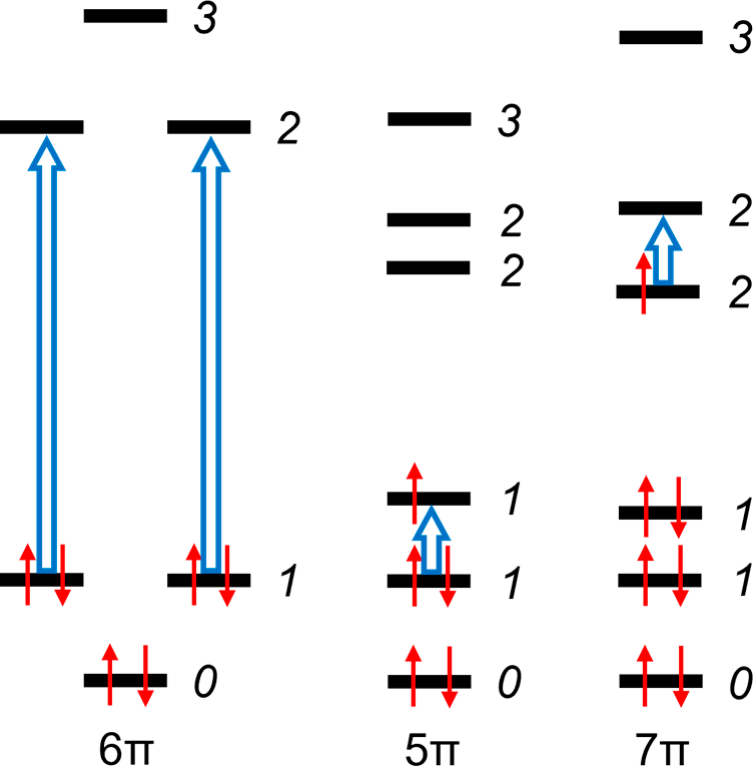
Schematic representation of Steiner and Fowler’s rule for 6π-aromatic systems and their radical ions. Red and blue arrows represent electrons and electronic transitions between the occupied and the virtual MOs. The number of nodal planes in the π system, perpendicular to the ring plane of the molecules, are written next to the energy levels.

Both the radical cation and the radical anion of furan sustain diatropic ring current. The radical anion of furan has a bent structure in which the spin density is mainly localized on oxygen and its two adjacent carbon atoms. In the radical cation of furan too, the spin density is mainly localized on the oxygen and its neighboring carbon atoms but the structure remains planar in its local minimum, Tables S3–S16. To better understand the variation of the current density in furan’s radical ions, the topology of their current density is compared with those of benzene radical ions, Figures 3 and 4. Furthermore, the current density of furan and its radical ions at various distances from their ring planes is depicted in Figure S3. In benzene, the π-ring current^37,38^ appears only when an isosurface value of 0.0007 au is chosen. This current is stronger in the outer rim of the ring, away from the C–C bonds. The ring current is the strongest in benzene’s radical anion based on both the topology of the current intensity and its isosurfaces and the integrated values, Table 1. The current density of the radical cation and anion have the usual topology of magnetically antiaromatic species, as the current density is stronger inside the framework of systems than in outer space.

**Figure 3:**
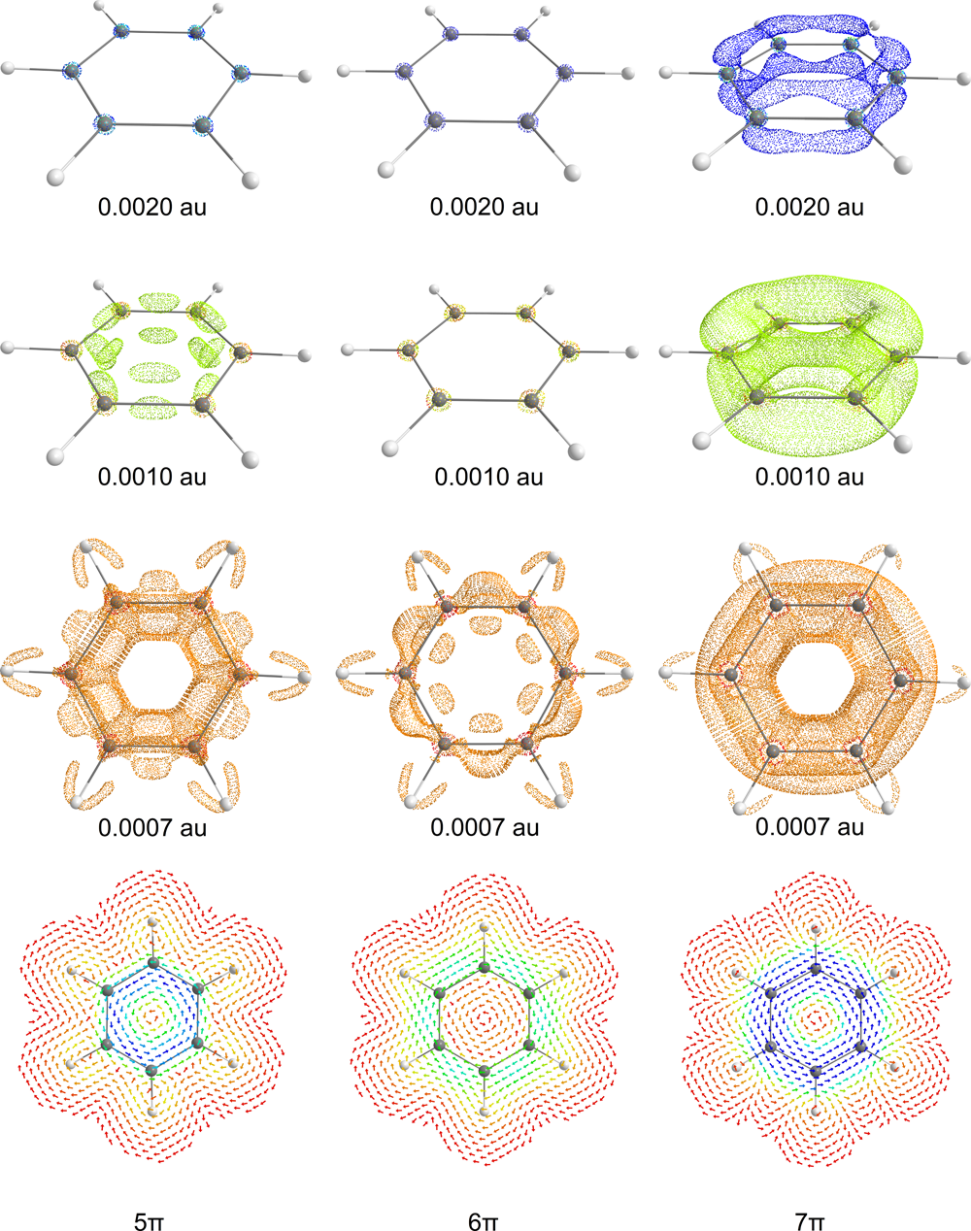
Isosurfaces of current intensity (both para- and diatropic) with selected values (0.0020, 0.0010, and 0.0007 au) plotted for benzene, its 5π-electron radical cation, and the 7π-electron radical anion. Furthermore, the profiles of current density, 1 Bohr above the ring plane, for benzene and its radical ions are presented. Blue to red denote strong (0.001 au) to weak (0.000 au) current intensities.

**Figure 4:**
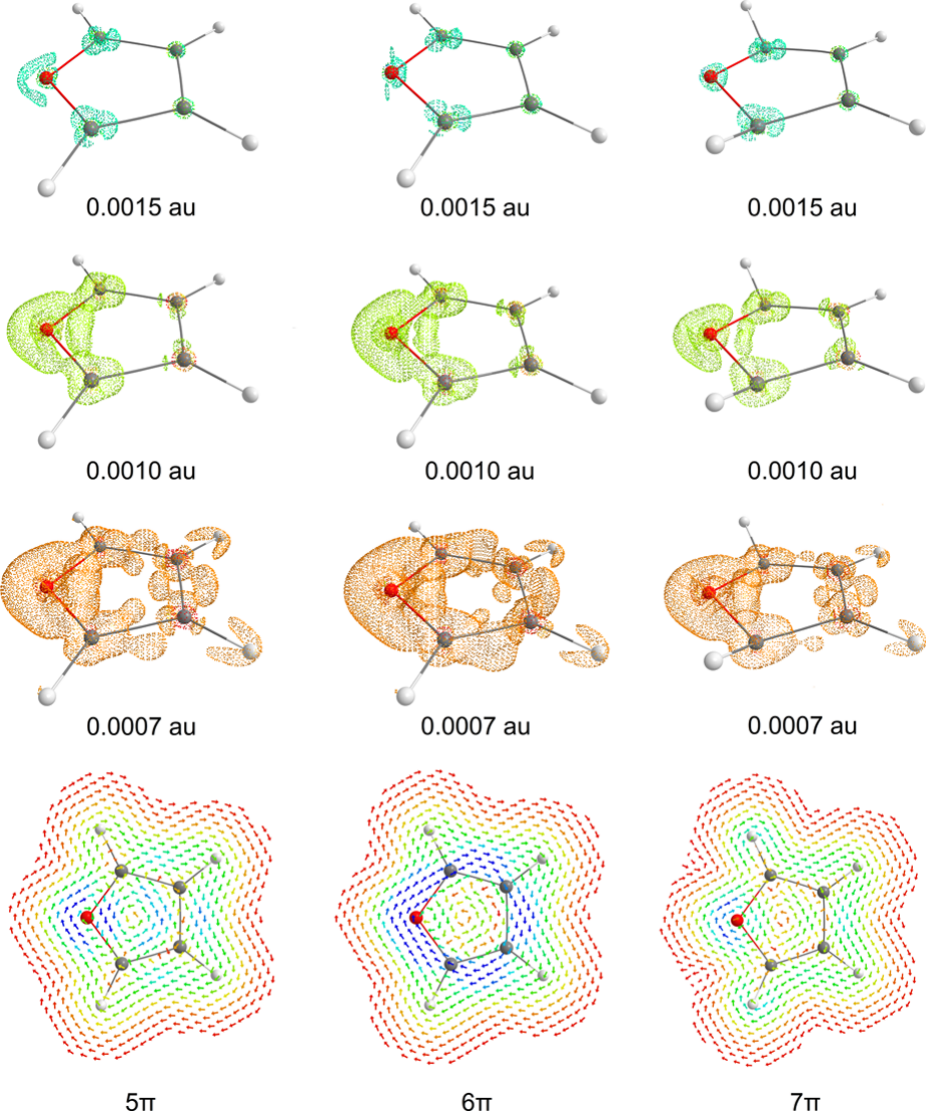
Isosurfaces of current intensity depicted (both para- and diatropic) with selected values (0.0015, 0.0010, and 0.0007 au) for furan, its 5π-electron radical cation, and the 7π-electron radical anion. Furthermore, the profiles of current density, 1 Bohr above the ring plane, for furan and its radical ions are presented. Blue to red denote strong (0.0005 au) to weak (0.0000 au) current intensities.

**Table 1:** Isomerization Stabilization Energies, ISEs,^a^ and Intensities of the Magnetically Induced Current Densities, MICDs,^b^ for the Parent 6π Molecules in Comparison with Their 7π Radical Anions and 5π Radical Cations

molecules		benzene	pyridine	1,2-diazine	1,3-diazine	1,4-diazine	pyrrole	furan
6π*e*	ISE	33.5	32.5	34.0	32.1	32.6	21.6	14.9
	MICD	12.0	11.5	11.0	10.7	11.2	11.5	10.2
7π*e*	ISE	6.7	8.0	11.2	6.4	10.7	c	–3.7
	MICD	–47.4	–25.0	–15.7	–22.6	–13.9	12.4^d^	24.0
5π*e*	ISE	7.4	c	29.4^c^	c	21.6^d^	1.0	0.0
	MICD	–17.3	11.9^d^	12.3^d^	10.7^d^	14.8^d^	–17.2	12.0

a ISE in kcal/mol.

b MICD in nA/T.

c Two molecules involved in the ISE equation do not have an equal number of π electrons because the π system of the nonaromatic reference system is reduced/oxidized.

d The system has 6π electrons. The electron is removed from the σ framework of the molecule.

The current density of furan’s radical ions has a weak π characteristic, unlike the parent compound, furan. It seems that the strong diatropicity in furan’s radical ions originates mainly from the sigma framework of the molecule as the profiles of the current density show a stronger diatropic π-ring current 1 Bohr above the ring plane of furan compared to its radical ions.

While both the radical anion and the radical cation of benzene sustain strong net paratropic ring current (−47.4 and −17.3 nA/T), which is a sign of magnetic antiaromaticity, the isomerization stabilization energies^39^ suggest that the radical ions are merely nonaromatic species that are even partially stabilized through cyclic conjugation compared to their acyclic counterparts. The isomerization stabilization energies of the radical anions of pyridine and diazines (**3**–**5**) similarly denote stabilization through cyclic conjugation. The most stabilized radical anion is that of 1,2-diazine with an ISE of 11.2 kcal/mol. The radical anions of pyridine and diazines, like that of benzene, all sustain strong paratropic ring currents that are inconsistent with the stabilization through cyclic conjugation.

The isomerization stabilization energies of the radical cations of 1,2- and 1,4-diazine denote considerable stabilization that is expected for a 6π-electron species. It is worth noting that ISE values for the radical cations of pyridine, 1,3-diazine, and the radical anion of pyrrole are not computable via comparison of the isomerization energies of the methylated derivatives. This is because unlike cyclic (aromatic) systems that sustain their 6π electrons, oxidation/reduction affects the electron population of the π framework of the acyclic (nonaromatic) reference systems. The radical cation of pyrrole has the lowest stabilization energy among the studied species consistent with a nonaromatic system without a notable conjugative stabilization. Radical ions of furan behave differently from those of the rest of the molecules. While the furan radical ions sustain a net diatropic ring current, the radicals are not stabilized according to the isomerization reaction. The origin of this behavior needs further in-depth analysis that is beyond the current report. The reported discrepancies between the ring current and the ISEs to the best of my knowledge have never been reported for benzene monocyclic heterocycles.

In summary, my results once again question the alleged relationship between magnetic and energetic criteria of aromaticity, this time magnetic antiaromaticity and energetic stability. As others have shown before,^14−16,19,21^ magnetic (anti)aromaticity, i.e., diatropic/paratropic ring current or a negative NICS^40^ value, is not necessarily a sign of stability or instability. However, this phenomenon has never been observed in the case of monocyclic aromatic hydrocarbons and small heterocycles. In particular, I showed that benzene, which is the prototype of aromatic compounds, can change its ring current from diatropic to paratropic upon one-electron reduction or oxidation, whereas radical ions of benzene both are stabilized by cyclic conjugation and cannot be classified as antiaromatic. The paratropic ring currents here are the result of orbital symmetries but not a sign of antiaromaticity, i.e., destabilization, because while energetic tools suggest the radical ions to be still stabilized, ring current suggests that they are antiaromatic. Nevertheless, the results of the present work question the validity of the paratropic ring current as a tool for the characterization of antiaromatic species.

## Data Availability

The data underlying this study are available in the published article and its Supporting Information.

## Supporting Information Available

Computational methods, plots of spin density, current density profiles of furan and its radical ions at different distances from the ring plane, spin density of all species on the atoms in the molecules, and Cartesian coordinates of all molecules computed at B3LYP/def2-TZVPP level (PDF)
