# Supplementary material for: Magnetic Antiaromaticity—Paratropicity—Does Not Necessarily Imply Instability
Source: J Org Chem. 2023 Sep 29;88(20):14831–5. doi: 10.1021/acs.joc.3c01807 (PMC10594649; doi:10.1021/acs.joc.3c01807)
Supplement: Supplementary file 1 — jo3c01807_si_001.pdf [file jo3c01807_si_001.pdf]

Supporting Information

Magnetic Antiaromaticity – Paratropicity – Does Not Necessarily Imply Instability

Cina Foroutan-Nejad*

Institute of Organic Chemistry, Polish Academy of Sciences, Kasprzaka 44/52, 01-224, Warsaw, Poland

Corresponding author's e-mail: cina.foroutan-nejad@icho.edu.pl

Computational Methods……………………………………………………………Page S3-S5

Figure S2…………………………………………………………………………Page S6

Figure S3…………………………………………………………………………Page S7

Spin Densities (Tables S3-S16.……………………………………………………..Page S8-S114

Cartesian Coordinates……………………………………………………………….Page S15-S34

Computational Methods

All structures are optimized at UB3LYP[1–4]/def2-TZVPP[5,6] computational level using a superfine integration grid. All structures coincide with the local minima based on the eigenvalues of the Hessian matrix. The potential multireference character of the radical ions was examined by the T1 diagnostic test at CCSD/def2-TZVP computational level. The T1 values,[7] listed in **Table S1**, were found to be notably less than the 0.044 threshold, recommended by Schaefer and his coworkers[8], for multireference characteristics of open-shell systems.[9] Therefore, DFT results were found to be reliable.

Table S1. T1 diagnostic test at CCSD/def2TZVP level for studied systems.

Molecules	**1**	**2**	**3**	**4**	**5**	**6**	**7**
$7\pi e$	0.0175	0.0212	0.0218	0.0234	0.0171	--------	0.0258
$5\pi e$	0.0194	--------	--------	--------	--------	0.0153	0.0192

The GIAO NMR computations were performed and WFX files were saved directly via Gaussian 16 C01 package.[10] The wavefunctions were analyzed via the AIMAll[11] suite of programs. To ensure that the studied radical anions and cations accommodate their electrons in their π-orbitals in their local minima, the electronic structures of all systems were analyzed. The current intensities are automatically generated via AIMAll as the flux of current density passing through the interatomic surfaces once a magnetic field is applied perpendicular to the ring plane of the molecules. The structures of the radical ions lose their symmetry mainly due to the Jahn-Teller distortion. As mentioned in the main text, a recent study concluded that M11[12] functional is a more reliable tool for assessing antiaromaticity. The ring current intensities computed at M11/def2-TZVPP level are listed in **Table S2**. The overall trends were found to be the same as MICDs computed at B3LYP/def2-TZVPP level however the absolute values of MICDs at M11 level are larger than their B3LYP counterparts. In order to make sure that the employed basis set size is enough, I computed MICD of benzene anion at B3LYP and M11 DFT levels by a large def2-QZVPPD basis set. The MICD values were found to be -47.6 and -66.4 nA/T, respectively. These values are essentially the same as the values computed by def2-TZVPP basis set within the error range of integration for MICDs.

Table S2. The intensities of the magnetically induced current densities, MICD[a] for the 7π-radical anions and 5π-radical cations at M11/def2-TZVPP computational level.

Molecules		Benzene	Pyridine	1,2-diazine	1,3-diazine	1,4-diazine	Pyrrole	Furan
$7\pi e$	MICD	–66.6	–31.4	–18.1	–27.3	–16.8	12.5[b]	25.3
$5\pi e$	MICD	–12.4	12.4[b]	13.1[b]	11.3[b]	15.7[b]	–16.7	16.7

a. **MICD in nA/T**

b. **The system has 6π-electrons. The electron is removed from the σ-framework of the molecule.**

The energetic stability of the radical ions with 5π or 7π-electrons is computed via isomerization stabilization energies from the reaction presented in **Figure S1**. All structures involved in the reaction are also optimized at B3LYP/def2-TZVPP computational level and correspond to the local minimum according to the eigenvalues of the Hessian matrix. ISEs are corrected for zero-point

vibrational energies. NBO[13] analysis was performed on the model systems used for assessing the ISE to verify that the systems have an equal number of π-electrons similar to the parent compounds.

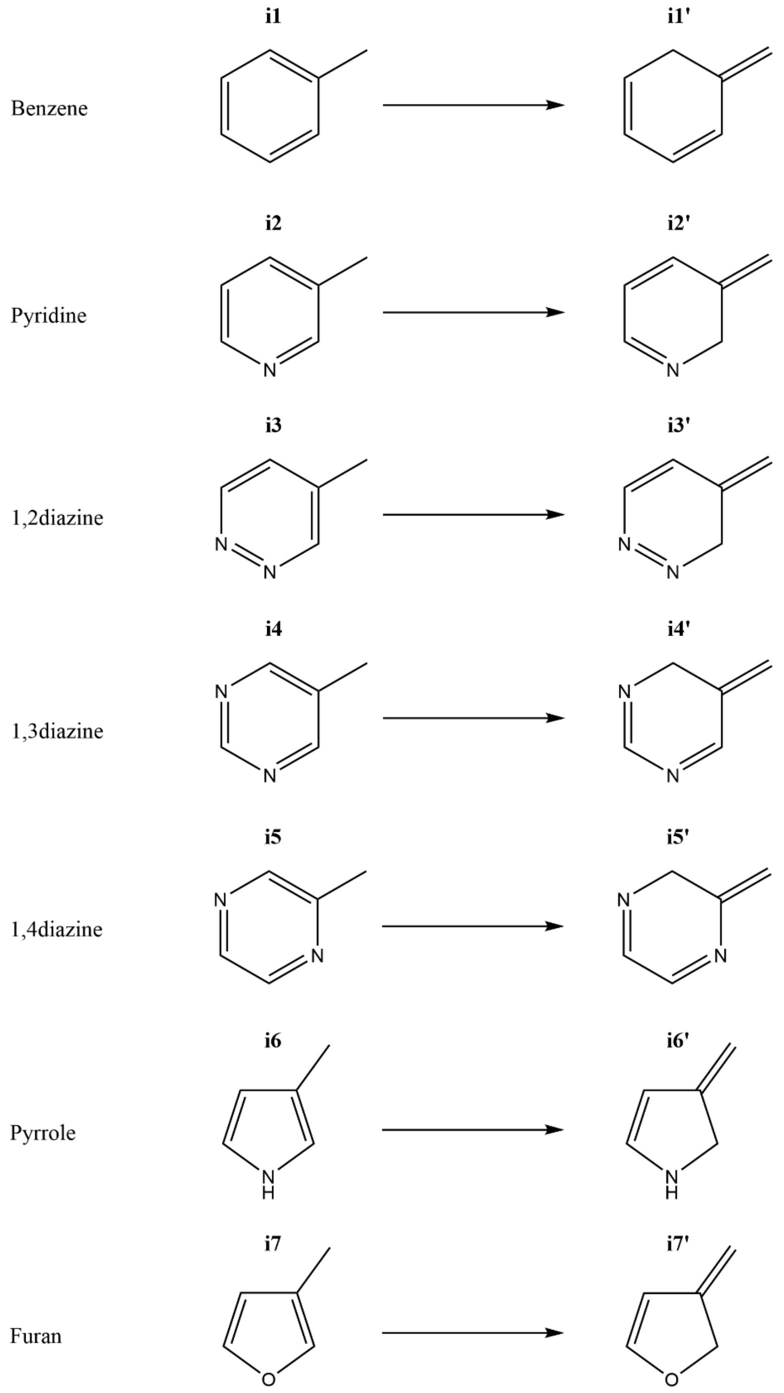

Figure S1. The ISEs were evaluated via the following reactions, **Figure S1** by comparison of the energies of (***in***) and (***in'***) molecules. The stabilizations of the radical ions of each system are also evaluated through the same model systems but with +1 or -1 charges, e.g., ***in***(+/-), and ***in'***(+/-), ***n*** = 1 – 7. The cartesian coordinates of the model systems are listed below.

References

(1) Becke, A. D. Density-Functional Exchange-Energy Approximation with Correct Asymptotic Behavior. *Phys. Rev. A* **1988**, *38* (6), 3098–3100. https://doi.org/10.1103/PhysRevA.38.3098.

(2) Becke, A. D. Density-functional Thermochemistry. III. The Role of Exact Exchange. *J. Chem. Phys.* **1993**, *98* (7), 5648–5652. https://doi.org/10.1063/1.464913.

(3) Lee, C.; Yang, W.; Parr, R. G. Development of the Colle-Salvetti Correlation-Energy Formula into a Functional of the Electron Density. *Phys. Rev. B* **1988**, *37* (2), 785–789. https://doi.org/10.1103/PhysRevB.37.785.

(4) Miehlich, B.; Savin, A.; Stoll, H.; Preuss, H. Results Obtained with the Correlation Energy Density Functionals of Becke and Lee, Yang and Parr. *Chem. Phys. Lett.* **1989**, *157* (3), 200–206. https://doi.org/10.1016/0009-2614(89)87234-3.

(5) Weigend, F.; Ahlrichs, R. Balanced Basis Sets of Split Valence, Triple Zeta Valence and Quadruple Zeta Valence Quality for H to Rn: Design and Assessment of Accuracy. *Phys. Chem. Chem. Phys.* **2005**, *7*, 3297–3305. https://doi.org/10.1039/B508541A.

(6) Weigend, F. Accurate Coulomb-Fitting Basis Sets for H to Rn. *Phys. Chem. Chem. Phys.* **2006**, *8* (9), 1057–1065. https://doi.org/10.1039/B515623H.

(7) Lee, T. J.; Taylor, P. R. A Diagnostic for Determining the Quality of Single-Reference Electron Correlation Methods. *Int. J. Quantum Chem.* **1989**, *36* (S23), 199–207. https://doi.org/10.1002/qua.560360824.

(8) Rienstra-Kiracofe, J. C.; Allen, W. D.; Schaefer, H. F. The C2H5 + O2 Reaction Mechanism: High-Level Ab Initio Characterizations. *J. Phys. Chem. A* **2000**, *104* (44), 9823–9840. https://doi.org/10.1021/jp001041k.

(9) Jayatilaka, D.; Lee, T. J. Open-shell Coupled-cluster Theory. *J. Chem. Phys.* **1993**, *98* (12), 9734–9747. https://doi.org/10.1063/1.464352.

(10) Frisch, M. J.; Trucks, G. W.; Schlegel, H. B.; Scuseria, G. E.; Robb, M. A.; Cheeseman, J. R.; Scalmani, G.; Barone, V.; Petersson, G. A.; Nakatsuji, H.; Li, X.; Caricato, M.; Marenich, A. V.; Bloino, J.; Janesko, B. G.; Gomperts, R.; Mennucci, B.; Hratchian, H. P.; Ortiz, J. V.; Izmaylov, A. F.; Sonnenberg, J. L.; Williams-Young, D.; Ding, F.; Lipparini, F.; Egidi, F.; Goings, J.; Peng, B.; Petrone, A.; Henderson, T.; Ranasinghe, D.; Zakrzewski, V. G.; Gao, J.; Rega, N.; Zheng, G.; Liang, W.; Hada, M.; Ehara, M.; Toyota, K.; Fukuda, R.; Hasegawa, J.; Ishida, M.; Nakajima, T.; Honda, Y.; Kitao, O.; Nakai, H.; Vreven, T.; Throssell, K.; Montgomery, Jr., J. A.; Peralta, J. E.; Ogliaro, F.; Bearpark, M. J.; Heyd, J. J.; Brothers, E. N.; Kudin, K. N.; Staroverov, V. N.; Keith, T. A.; Kobayashi, R.; Normand, j.; Raghavachari, K.; Rendell, A. P.; Burant, J. C.; Iyengar, S. S.; Tomasi, J.; Cossi, M.; Millam, J. M.; Klene, M.; Adamo, C.; Cammi, R.; Ochterski, J. W.; Martin, R. L.; Morokuma, K.; Farkas, O.; Foresman, J. B.; Fox, D. J. Gaussian 16, 2016.

(11) Keith, T. A. AIMAll 19.10.12 (Aim.Tkgristmill.Com), 2019. aim.tkgristmill.com.

(12) Peverati, R.; Truhlar, D. G. Improving the Accuracy of Hybrid Meta-GGA Density Functionals by Range Separation. *J. Phys. Chem. Lett.* **2011**, *2* (21), 2810–2817. https://doi.org/10.1021/jz201170d.

(13) Landis, C. R.; Weinhold, F. The NBO View of Chemical Bonding. In *The Chemical Bond*; John Wiley & Sons, Ltd, 2014; pp 91–120. https://doi.org/10.1002/9783527664696.ch3.

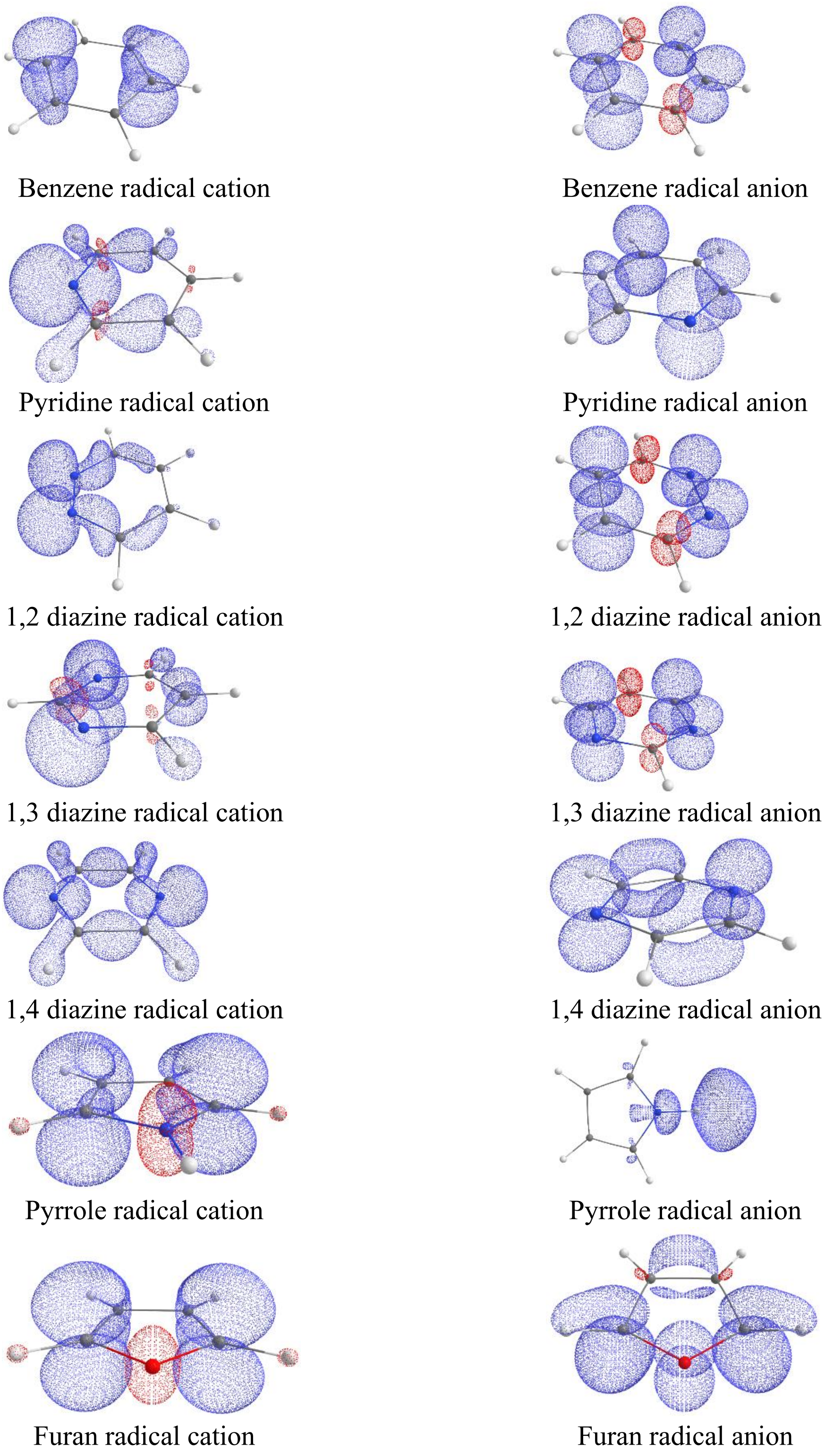

Figure S2. Spin density plots of all studied species.

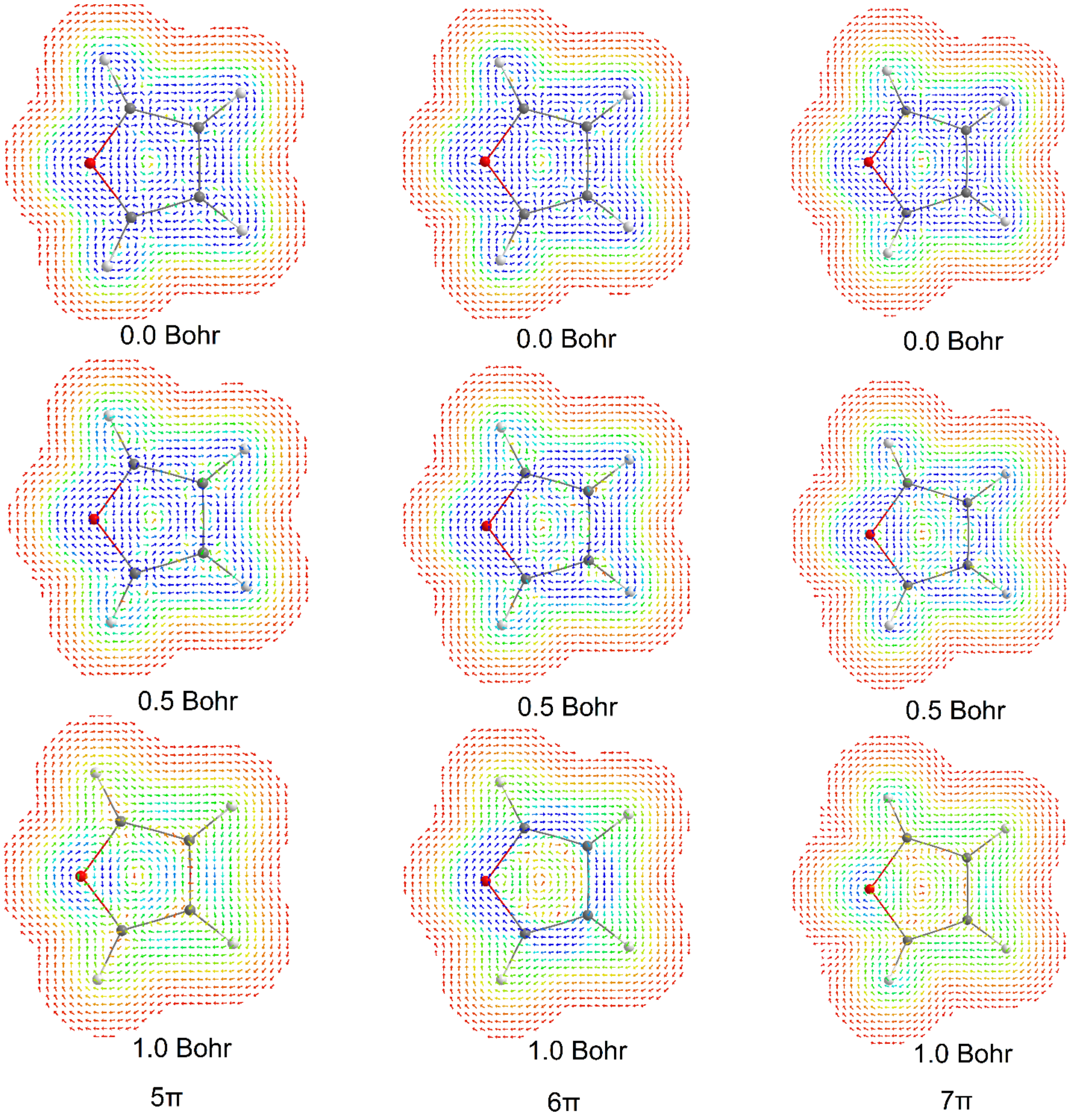

Figure S3. The ring current plots for 5π, 6π, and 7π electron furan radical cation, furan, and furan radical anion on the ring plane, 0.5 bohr above the ring plane, and 1 bohr above the ring plane of the molecules. Dark blue to red represents high to low ring current intensities.

Spin population of atoms in molecules for the studied systems

Table S3. Bnzene radical cation:

Atom A	N_alpha(A)	N_beta(A)	N_total(A)	N_spin(A)
C1	3.0094615656E+00	2.9362674206E+00	5.9457289861E+00	7.3194145008E-02
C2	2.9803662999E+00	2.9607354025E+00	5.9411017024E+00	1.9630897349E-02
C3	3.1799919308E+00	2.7715301507E+00	5.9515220816E+00	4.0846178006E-01
C4	3.0094124355E+00	2.9362183590E+00	5.9456307945E+00	7.3194076537E-02
C5	2.9803520534E+00	2.9607244011E+00	5.9410764545E+00	1.9627652316E-02
C6	3.1799824577E+00	2.7715175783E+00	5.9515000360E+00	4.0846487937E-01
H7	4.4507575068E-01	4.4562656985E-01	8.9070232053E-01	-5.5081917259E-04
H8	4.4607397438E-01	4.4671946614E-01	8.9279344052E-01	-6.4549175509E-04
H9	4.3907075068E-01	4.3915845606E-01	8.7822920675E-01	-8.7705378623E-05
H10	4.4507576087E-01	4.4562632287E-01	8.9070208374E-01	-5.5056199993E-04
H11	4.4607386997E-01	4.4671962674E-01	8.9279349671E-01	-6.4575677066E-04
H12	4.3907339827E-01	4.3916133893E-01	8.7823473720E-01	-8.7940654902E-05
Total	2.1000010248E+01	2.0000005093E+01	4.1000015341E+01	1.0000051549E+00

Table S4. Benzene radical anion:

Atom A	N_alpha(A)	N_beta(A)	N_total(A)	N_spin(A)
C1	3.1788964930E+00	2.9264188441E+00	6.1053153371E+00	2.5247764890E-01
C2	3.0027706770E+00	3.0346453627E+00	6.0374160397E+00	-3.1874685627E-02
C3	3.1788976526E+00	2.9264065761E+00	6.1053042286E+00	2.5249107647E-01
C4	3.1788669174E+00	2.9263978501E+00	6.1052647675E+00	2.5246906726E-01
C5	3.0028056823E+00	3.0346818574E+00	6.0374875397E+00	-3.1876175017E-02
C6	3.1789161020E+00	2.9264205686E+00	6.1053366705E+00	2.5249553341E-01
H7	5.5043822536E-01	5.3673998246E-01	1.0871782078E+00	1.3698242898E-02
H8	5.3856873642E-01	5.3904736392E-01	1.0776161003E+00	-4.7862750110E-04
H9	5.5044314710E-01	5.3674245245E-01	1.0871855996E+00	1.3700694654E-02
H10	5.5044173463E-01	5.3674172534E-01	1.0871834600E+00	1.3700009294E-02
H11	5.3856892733E-01	5.3904749338E-01	1.0776164207E+00	-4.7856604788E-04
H12	5.5043848661E-01	5.3674077848E-01	1.0871792651E+00	1.3697708136E-02
Total	2.2000052782E+01	2.1000030855E+01	4.3000083637E+01	1.0000219268E+00

Table S5. Pyridine radical cation:

Atom A	N_alpha(A)	N_beta(A)	N_total(A)	N_spin(A)
C1	2.9551549421E+00	2.9822190788E+00	5.9373740209E+00	-2.7064136777E-02
C2	3.0061600719E+00	2.9328105604E+00	5.9389706324E+00	7.3349511522E-02
C3	2.7289863295E+00	2.7199680373E+00	5.4489543668E+00	9.0182922130E-03
C4	2.7290713985E+00	2.7200534928E+00	5.4491248913E+00	9.0179057043E-03
C5	3.0061757365E+00	2.9328261593E+00	5.9390018958E+00	7.3349577216E-02
H6	4.4596773124E-01	4.3898440792E-01	8.8495213917E-01	6.9833233225E-03
H7	4.4457591965E-01	4.3062830432E-01	8.7520422397E-01	1.3947615335E-02
H8	4.2765215259E-01	3.9819494901E-01	8.2584710160E-01	2.9457203585E-02
H9	4.2765067501E-01	3.9819344865E-01	8.2584412366E-01	2.9457226364E-02
H10	4.4457806327E-01	4.3063048020E-01	8.7520854347E-01	1.3947583073E-02
N11	4.3840714829E+00	3.6155384395E+00	7.9996099225E+00	7.6853304336E-01
Total	2.1000044503E+01	2.0000047358E+01	4.1000091862E+01	9.9999714492E-01

Table S6. Pyridine radical anion:

Atom A	N_alpha(A)	N_beta(A)	N_total(A)	N_spin(A)
C1	3.2543560498E+00	2.8677122861E+00	6.1220683359E+00	3.8664376365E-01
C2	3.0549082058E+00	3.0127777080E+00	6.0676859138E+00	4.2130497758E-02
C3	2.8619316740E+00	2.7745719781E+00	5.6365036521E+00	8.7359695874E-02
C4	2.8618047209E+00	2.7744755190E+00	5.6362802399E+00	8.7329201971E-02
C5	3.0548405407E+00	3.0127223367E+00	6.0675628774E+00	4.2118204071E-02
H6	5.5033832825E-01	5.2990607804E-01	1.0802444063E+00	2.0432250205E-02
H7	5.3878465617E-01	5.3594011591E-01	1.0747247721E+00	2.8445402556E-03
H8	5.3934798611E-01	5.3588782305E-01	1.0752358092E+00	3.4601630634E-03
H9	5.3934629996E-01	5.3588729328E-01	1.0752335932E+00	3.4590066732E-03
H10	5.3878572433E-01	5.3594191527E-01	1.0747276396E+00	2.8438090640E-03
N11	4.2054665753E+00	3.8841249375E+00	8.0895915128E+00	3.2134163775E-01
Total	2.1999910761E+01	2.0999947991E+01	4.2999858752E+01	9.9996277034E-01

Table S7. 1,2 diazine radical cation:

Atom A	N_alpha(A)	N_beta(A)	N_total(A)	N_spin(A)
C1	2.9715379754E+00	2.9608350412E+00	5.9323730166E+00	1.0702934192E-02
C2	2.7495290547E+00	2.7097253402E+00	5.4592543949E+00	3.9803714530E-02
C3	2.7495591170E+00	2.7097539659E+00	5.4593130829E+00	3.9805151148E-02
C4	2.9714915562E+00	2.9607878229E+00	5.9322793790E+00	1.0703733307E-02
H5	4.3823938686E-01	4.2557426672E-01	8.6381365358E-01	1.2665120138E-02
H6	4.1644487496E-01	4.1851828752E-01	8.3496316248E-01	-2.0734125572E-03
H7	4.1644326361E-01	4.1851662967E-01	8.3495989329E-01	-2.0733660632E-03
H8	4.3823949304E-01	4.2557434032E-01	8.6381383336E-01	1.2665152717E-02
N9	3.9242742395E+00	3.4853740375E+00	7.4096482770E+00	4.3890020197E-01
N10	3.9242804451E+00	3.4853817318E+00	7.4096621769E+00	4.3889871327E-01
Total	2.1000039406E+01	2.0000041464E+01	4.1000080870E+01	9.9999794265E-01

Table S8. 1,2 diazine radical anion:

Atom A	N_alpha(A)	N_beta(A)	N_total(A)	N_spin(A)
C1	3.1712324054E+00	2.9302038077E+00	6.1014362130E+00	2.4102859770E-01
C2	2.7472220124E+00	2.7861734191E+00	5.5333954316E+00	-3.8951406668E-02
C3	2.7471472515E+00	2.7861109352E+00	5.5332581866E+00	-3.8963683683E-02
C4	3.1710638188E+00	2.9300446804E+00	6.1011084992E+00	2.4101913844E-01
H5	5.4057283130E-01	5.2868981777E-01	1.0692626491E+00	1.1883013531E-02
H6	5.3085142719E-01	5.3222532543E-01	1.0630767526E+00	-1.3738982426E-03
H7	5.3085111243E-01	5.3222505066E-01	1.0630761631E+00	-1.3739382289E-03
H8	5.4057188938E-01	5.2868911231E-01	1.0692610017E+00	1.1882777068E-02
N9	4.0102164312E+00	3.7228017708E+00	7.7330182020E+00	2.8741466037E-01
N10	4.0102478555E+00	3.7228336515E+00	7.7330815070E+00	2.8741420395E-01
Total	2.1999977035E+01	2.0999997571E+01	4.2999974606E+01	9.9997946424E-01

Table S9. 1,3 diazine radical cation:

Atom A	N_alpha(A)	N_beta(A)	N_total(A)	N_spin(A)
C1	2.4283228128E+00	2.4481801694E+00	4.8765029822E+00	-1.9857356544E-02
C2	2.6946716647E+00	2.7061555699E+00	5.4008272345E+00	-1.1483905200E-02
C3	2.9984889218E+00	2.9241186860E+00	5.9226076078E+00	7.4370235784E-02
C4	2.6946679622E+00	2.7061522292E+00	5.4008201914E+00	-1.1484267043E-02
H5	4.0525916448E-01	4.0898467087E-01	8.1424383535E-01	-3.7255063945E-03
H6	4.3168036946E-01	4.0478440356E-01	8.3646477302E-01	2.6895965900E-02
H7	4.3128786078E-01	4.3364838223E-01	8.6493624301E-01	-2.3605214496E-03
H8	4.3167767374E-01	4.0478178294E-01	8.3645945668E-01	2.6895890799E-02
N9	4.2419622348E+00	3.7815867133E+00	8.0235489481E+00	4.6037552157E-01
N10	4.2419512644E+00	3.7815740302E+00	8.0235252946E+00	4.6037723424E-01
Total	2.0999969929E+01	1.9999966638E+01	4.0999936567E+01	1.0000032917E+00

Table S10. 1,3 diazine radical anion:

Atom A	N_alpha(A)	N_beta(A)	N_total(A)	N_spin(A)
C1	2.4653144347E+00	2.5026934882E+00	4.9680079230E+00	-3.7379053514E-02
C2	3.0210426682E+00	2.6983636428E+00	5.7194063109E+00	3.2267902541E-01
C3	2.9990556411E+00	3.0499310256E+00	6.0489866667E+00	-5.0875384423E-02
C4	3.0211311272E+00	2.6984592405E+00	5.7195903678E+00	3.2267188669E-01
H5	5.3057162608E-01	5.3274102740E-01	1.0633126535E+00	-2.1694013252E-03
H6	5.4102653895E-01	5.2741005324E-01	1.0684365922E+00	1.3616485708E-02
H7	5.3154711061E-01	5.3308157302E-01	1.0646286836E+00	-1.5344624156E-03
H8	5.4102394321E-01	5.2740734880E-01	1.0684312920E+00	1.3616594407E-02
N9	4.1746076967E+00	3.9649226043E+00	8.1395303010E+00	2.0968509234E-01
N10	4.1745961088E+00	3.9649095416E+00	8.1395056505E+00	2.0968656720E-01
Total	2.1999916896E+01	2.0999919545E+01	4.2999836441E+01	9.9999735008E-01

Table S11. 1,4 diazine radical cation:

Atom A	N_alpha(A)	N_beta(A)	N_total(A)	N_spin(A)
C1	2.7102430928E+00	2.6615900006E+00	5.3718330934E+00	4.8653092150E-02
C2	2.7102195703E+00	2.6615666416E+00	5.3717862119E+00	4.8652928781E-02
C3	2.7101890409E+00	2.6615368618E+00	5.3717259027E+00	4.8652179060E-02
C4	2.7102147417E+00	2.6615623708E+00	5.3717771125E+00	4.8652370922E-02
H5	4.2851338808E-01	3.9673068732E-01	8.2524407540E-01	3.1782700761E-02
H6	4.2851360726E-01	3.9673031020E-01	8.2524391747E-01	3.1783297057E-02
H7	4.2851434283E-01	3.9673095697E-01	8.2524529981E-01	3.1783385864E-02
H8	4.2851267530E-01	3.9672998794E-01	8.2524266325E-01	3.1782687357E-02
N9	4.2225356536E+00	3.8834071481E+00	8.1059428017E+00	3.3912850556E-01
N10	4.2225359265E+00	3.8834070476E+00	8.1059429742E+00	3.3912887890E-01
Total	2.0999992039E+01	1.9999992013E+01	4.0999984052E+01	1.0000000264E+00

Table S12. 1,4 diazine radical anion:

Atom A	N_alpha(A)	N_beta(A)	N_total(A)	N_spin(A)
C1	2.8564752826E+00	2.7865731557E+00	5.6430484383E+00	6.9902126865E-02
C2	2.8564691958E+00	2.7865744022E+00	5.6430435980E+00	6.9894793651E-02
C3	2.8565335401E+00	2.7866199842E+00	5.6431535243E+00	6.9913555968E-02
C4	2.8565055701E+00	2.7865845787E+00	5.6430901488E+00	6.9920991434E-02
H5	5.3546307947E-01	5.3316965716E-01	1.0686327366E+00	2.2934223143E-03
H6	5.3546269944E-01	5.3317000741E-01	1.0686327069E+00	2.2926920277E-03
H7	5.3546339278E-01	5.3317016411E-01	1.0686335569E+00	2.2932286685E-03
H8	5.3546372429E-01	5.3317009385E-01	1.0686338181E+00	2.2936304379E-03
N9	4.2160263810E+00	3.8604365166E+00	8.0764628976E+00	3.5558986438E-01
N10	4.2160237193E+00	3.8604346114E+00	8.0764583307E+00	3.5558910788E-01
Total	2.1999886585E+01	2.0999903171E+01	4.2999789756E+01	9.9998341363E-01

Table S13. Pyrrole radical cation:

Atom A	N_alpha(A)	N_beta(A)	N_total(A)	N_spin(A)
C1	2.9685373387E+00	2.5358135121E+00	5.5043508509E+00	4.3272382659E-01
C2	3.0071071725E+00	2.9345979508E+00	5.9417051234E+00	7.2509221671E-02
C3	3.0071266481E+00	2.9346189303E+00	5.9417455784E+00	7.2507717769E-02
C4	2.9685338604E+00	2.5358074378E+00	5.5043412982E+00	4.3272642265E-01
N5	4.0924241667E+00	4.0993857166E+00	8.1918098833E+00	-6.9615499794E-03
H6	2.5165036695E-01	2.5135320439E-01	5.0300357134E-01	2.9716255677E-04
H7	4.1902670589E-01	4.2005655936E-01	8.3908326525E-01	-1.0298534652E-03
H8	4.3331650541E-01	4.3418840008E-01	8.6750490548E-01	-8.7189467195E-04
H9	4.3331686245E-01	4.3418869150E-01	8.6750555395E-01	-8.7182905650E-04
H10	4.1902957479E-01	4.2005953249E-01	8.3908910727E-01	-1.0299576982E-03
Total	1.8000069202E+01	1.7000069935E+01	3.5000139137E+01	9.9999926637E-01

Table S14. Pyrrole radical anion:

Atom A	N_alpha(A)	N_beta(A)	N_total(A)	N_spin(A)
C1	2.8258524017E+00	2.8059387725E+00	5.6317911742E+00	1.9913629191E-02
C2	3.0423935480E+00	3.0379935392E+00	6.0803870872E+00	4.4000087778E-03
C3	3.0422858304E+00	3.0379042726E+00	6.0801901029E+00	4.3815577865E-03
C4	2.8258728794E+00	2.8059337311E+00	5.6318066105E+00	1.9939148268E-02
N5	4.1846505271E+00	4.0282223551E+00	8.2128728822E+00	1.5642817200E-01
H6	8.7730393713E-01	2.6383851121E-01	1.1411424483E+00	6.1346542592E-01
H7	5.5668052636E-01	4.9202279802E-01	1.0487033244E+00	6.4657728347E-02
H8	5.4406269130E-01	5.1806213625E-01	1.0621248276E+00	2.6000555051E-02
H9	5.4406191714E-01	5.1806131355E-01	1.0621232307E+00	2.6000603591E-02
H10	5.5668244328E-01	4.9202553751E-01	1.0487079808E+00	6.4656905763E-02
Total	1.8999846702E+01	1.8000002967E+01	3.6999849669E+01	9.9984373470E-01

Table S15. Furan radical cation:

Atom A	N_alpha(A)	N_beta(A)	N_total(A)	N_spin(A)
C1	2.9138654150E+00	2.4885697265E+00	5.4024351414E+00	4.2529568849E-01
C2	2.9972646713E+00	2.9275944416E+00	5.9248591129E+00	6.9670229781E-02
C3	2.9973261802E+00	2.9275602256E+00	5.9248864058E+00	6.9765954597E-02
C4	2.9137792251E+00	2.4885167982E+00	5.4022960233E+00	4.2526242694E-01
O5	4.5337084928E+00	4.5156466227E+00	9.0493551156E+00	1.8061870081E-02
H6	4.0035570205E-01	4.0336737743E-01	8.0372307948E-01	-3.0116753819E-03
H7	4.2167457013E-01	4.2269067448E-01	8.4436524460E-01	-1.0161043531E-03
H8	4.2167299788E-01	4.2268923345E-01	8.4436223133E-01	-1.0162355636E-03
H9	4.0035877105E-01	4.0337003406E-01	8.0372880511E-01	-3.0112630107E-03
Total	1.8000006026E+01	1.7000005134E+01	3.5000011160E+01	1.0000008916E+00

Table S16. Furan radical anion:

Atom A	N_alpha(A)	N_beta(A)	N_total(A)	N_spin(A)
C1	3.0406085240E+00	2.7073390866E+00	5.7479476106E+00	3.3326943743E-01
C2	3.0601293348E+00	3.0310342239E+00	6.0911635587E+00	2.9095110955E-02
C3	3.0600596621E+00	3.0309711005E+00	6.0910307626E+00	2.9088561622E-02
C4	3.0406335300E+00	2.7073889569E+00	5.7480224869E+00	3.3324457303E-01
O5	4.6119264845E+00	4.4316578045E+00	9.0435842890E+00	1.8026868002E-01
H6	5.5906777280E-01	5.1304632702E-01	1.0721140998E+00	4.6021445776E-02
H7	5.3423799155E-01	5.3274656086E-01	1.0669845524E+00	1.4914306891E-03
H8	5.3423715742E-01	5.3274617327E-01	1.0669833307E+00	1.4909841474E-03
H9	5.5906356767E-01	5.1304454146E-01	1.0721081091E+00	4.6019026215E-02
Total	1.8999964025E+01	1.7999974775E+01	3.6999938800E+01	9.9998924988E-01

Cartesian coordinates of all studied species at B3LYP/def2-TZVPP computational level

Benzene

6	1.204731000	0.695552000	0.000000000
6	0.000000000	1.391104000	0.000000000
6	-1.204731000	0.695552000	0.000000000
6	-1.204731000	-0.695552000	0.000000000
6	0.000000000	-1.391104000	0.000000000
6	1.204731000	-0.695552000	0.000000000
1	2.141940000	1.236650000	0.000000000
1	0.000000000	2.473300000	0.000000000
1	-2.141940000	1.236650000	0.000000000
1	-2.141940000	-1.236650000	0.000000000
1	0.000000000	-2.473300000	0.000000000
1	2.141940000	-1.236650000	0.000000000

Pyridine

6	1.227325000	0.708596000	0.000000000
6	0.016066000	1.387661000	0.000000000
6	-1.160114000	0.646031000	0.000000000
6	-0.020577000	-1.327703000	0.000000000
6	1.209782000	-0.679916000	0.000000000
1	2.164495000	1.249671000	0.000000000
1	-0.021401000	2.468535000	0.000000000
1	-2.122914000	1.145496000	0.000000000
1	-0.069428000	-2.411246000	0.000000000
1	2.127113000	-1.252802000	0.000000000
7	-1.189959000	-0.687024000	0.000000000

1,2-diazine

6	0.000000000	0.689002000	1.197070000
6	0.000000000	1.319192000	-0.044652000
6	0.000000000	-1.319192000	-0.044652000
6	0.000000000	-0.689002000	1.197070000
1	0.000000000	1.265460000	2.112157000
1	0.000000000	2.398957000	-0.128624000
1	0.000000000	-2.398957000	-0.128624000
1	0.000000000	-1.265460000	2.112157000
7	0.000000000	-0.663605000	-1.202299000
7	0.000000000	0.663605000	-1.202299000

1,3-diazine

6	0.000000000	0.000000000	1.296504000
6	0.000000000	1.180707000	-0.631177000
6	0.000000000	0.000000000	-1.360035000
6	0.000000000	-1.180707000	-0.631177000
1	0.000000000	0.000000000	2.380910000

1	0.000000000	2.146330000	-1.124973000
1	0.000000000	0.000000000	-2.440843000
1	0.000000000	-2.146330000	-1.124973000
7	0.000000000	1.191434000	0.701488000
7	0.000000000	-1.191434000	0.701488000

1,4-diazine

6	1.167602000	0.631041000	0.000000000
6	-0.037303000	1.326694000	0.000000000
6	-1.167602000	-0.631041000	0.000000000
6	0.037303000	-1.326694000	0.000000000
1	2.114546000	1.158701000	0.000000000
1	-0.053809000	2.410602000	0.000000000
1	-2.114546000	-1.158701000	0.000000000
1	0.053809000	-2.410602000	0.000000000
7	-1.211707000	0.699580000	0.000000000
7	1.211707000	-0.699580000	0.000000000

Pyrrole

6	0.000000000	-1.121756000	-0.325231000
6	0.000000000	-0.710676000	0.985673000
6	0.000000000	0.710676000	0.985673000
6	0.000000000	1.121756000	-0.325231000
7	0.000000000	0.000000000	-1.112939000
1	0.000000000	0.000000000	-2.116158000
1	0.000000000	-2.107473000	-0.756263000
1	0.000000000	-1.356199000	1.847460000
1	0.000000000	1.356199000	1.847460000
1	0.000000000	2.107473000	-0.756263000

Furan

6	-0.337382000	-1.094664000	0.000000000
6	0.962313000	-0.711810000	0.000000000
6	0.955693000	0.720633000	0.000000000
6	-0.347482000	1.091486000	0.000000000
8	-1.152620000	-0.005322000	0.000000000
1	-0.828327000	-2.050935000	0.000000000
1	1.820752000	-1.360934000	0.000000000
1	1.808106000	1.377647000	0.000000000
1	-0.847198000	2.043206000	0.000000000

Benzene radical cation:

6	1.208759000	0.737365000	0.000000000
6	0.027592000	1.422457000	0.000000000
6	-1.191117000	0.693454000	0.000000000
6	-1.208759000	-0.737365000	0.000000000
6	-0.027592000	-1.422457000	0.000000000

6	1.191117000	-0.693454000	0.000000000
1	2.158624000	1.253542000	0.000000000
1	0.003851000	2.502953000	0.000000000
1	-2.131670000	1.230303000	0.000000000
1	-2.158624000	-1.253542000	0.000000000
1	-0.003851000	-2.502953000	0.000000000
1	2.131670000	-1.230303000	0.000000000

Benzene radical anion:

6	1.223688000	0.728326000	0.000000000
6	0.000000000	1.394804000	0.000000000
6	-1.223688000	0.728326000	0.000000000
6	-1.223688000	-0.728326000	0.000000000
6	0.000000000	-1.394804000	0.000000000
6	1.223688000	-0.728326000	0.000000000
1	2.153959000	1.285154000	0.000000000
1	0.000000000	2.484313000	0.000000000
1	-2.153959000	1.285154000	0.000000000
1	-2.153959000	-1.285154000	0.000000000
1	0.000000000	-2.484313000	0.000000000
1	2.153959000	-1.285154000	0.000000000

Pyridine radical cation:

6	1.208242000	0.697579000	0.000000000
6	0.011109000	1.404386000	0.000000000
6	-1.193614000	0.691289000	0.000000000
6	0.001867000	-1.379344000	0.000000000
6	1.221789000	-0.692572000	0.000000000
1	2.145704000	1.238823000	0.000000000
1	-0.031530000	2.485086000	0.000000000
1	-2.181635000	1.136027000	0.000000000
1	-0.106990000	-2.457365000	0.000000000
1	2.136383000	-1.269849000	0.000000000
7	-1.050936000	-0.606759000	0.000000000

Pyridine radical anion:

6	1.270143000	0.733318000	0.000000000
6	-0.000108000	1.390681000	0.000000000
6	-1.160612000	0.657759000	0.000000000
6	-0.010669000	-1.333999000	0.000000000
6	1.204311000	-0.695433000	0.000000000
1	2.206789000	1.274089000	0.000000000
1	-0.063832000	2.476108000	0.000000000
1	-2.117298000	1.179905000	0.000000000
1	-0.036821000	-2.423587000	0.000000000
1	2.112454000	-1.293335000	0.000000000
7	-1.243968000	-0.718207000	0.000000000

1,2 diazine radical cation:

6	0.000000000	0.693566000	1.173929000
6	0.000000000	1.380315000	-0.042388000
6	0.000000000	-1.380315000	-0.042388000
6	0.000000000	-0.693566000	1.173929000
1	0.000000000	1.258232000	2.097232000
1	0.000000000	2.451981000	-0.168191000
1	0.000000000	-2.451981000	-0.168192000
1	0.000000000	-1.258232000	2.097232000
7	0.000000000	-0.606193000	-1.126930000
7	0.000000000	0.606193000	-1.126930000

1,2 diazine radical anion:

6	0.000000000	0.719158000	1.204963000
6	0.000000000	1.319973000	-0.043709000
6	0.000000000	-1.319973000	-0.043709000
6	0.000000000	-0.719158000	1.204963000
1	0.000000000	1.311039000	2.112005000
1	0.000000000	2.410165000	-0.109668000
1	0.000000000	-2.410165000	-0.109668000
1	0.000000000	-1.311039000	2.112005000
7	0.000000000	-0.709596000	-1.229938000
7	0.000000000	0.709596000	-1.229938000

1,3 diazine radical cation:

6	0.000000000	0.000000000	1.383700000
6	0.000000000	1.170698000	-0.651880000
6	0.000000000	0.000000000	-1.407596000
6	0.000000000	-1.170698000	-0.651880000
1	0.000000000	0.000000000	2.463415000
1	0.000000000	2.159167000	-1.102023000
1	0.000000000	0.000000000	-2.487640000
1	0.000000000	-2.159167000	-1.102023000
7	0.000000000	1.106945000	0.661569000
7	0.000000000	-1.106945000	0.661569000

1,3 diazine radical anion:

6	0.000000000	0.000000000	1.299052000
6	0.000000000	1.198225000	-0.656370000
6	0.000000000	0.000000000	-1.354894000
6	0.000000000	-1.198225000	-0.656370000
1	0.000000000	0.000000000	2.394407000
1	0.000000000	2.160663000	-1.155154000
1	0.000000000	0.000000000	-2.443225000
1	0.000000000	-2.160663000	-1.155154000
7	0.000000000	1.209618000	0.747460000

7	0.000000000	-1.209618000	0.747460000

1,4 diazine radical cation:

6	1.194707000	0.642129000	0.000000000
6	-0.041253000	1.355711000	0.000000000
6	-1.194707000	-0.642129000	0.000000000
6	0.041253000	-1.355711000	0.000000000
1	2.140550000	1.172640000	0.000000000
1	-0.054739000	2.440090000	0.000000000
1	-2.140550000	-1.172640000	0.000000000
1	0.054739000	-2.440090000	0.000000000
7	-1.136909000	0.656395000	0.000000000
7	1.136909000	-0.656395000	0.000000000

1,4 diazine radical anion:

6	1.159873000	0.638148000	0.000000000
6	-0.027284000	1.323554000	0.000000000
6	-1.159873000	-0.638148000	0.000000000
6	0.027284000	-1.323554000	0.000000000
1	2.095230000	1.197102000	0.000000000
1	-0.010895000	2.413074000	0.000000000
1	-2.095230000	-1.197102000	0.000000000
1	0.010895000	-2.413074000	0.000000000
7	-1.269732000	0.733081000	0.000000000
7	1.269732000	-0.733081000	0.000000000

Pyrrole radical cation:

6	0.000000000	-1.105965000	-0.346362000
6	0.000000000	-0.684165000	1.018922000
6	0.000000000	0.684165000	1.018922000
6	0.000000000	1.105965000	-0.346362000
7	0.000000000	0.000000000	-1.133979000
1	0.000000000	0.000000000	-2.144431000
1	0.000000000	-2.101246000	-0.763476000
1	0.000000000	-1.348012000	1.867211000
1	0.000000000	1.348012000	1.867211000
1	0.000000000	2.101246000	-0.763476000

Pyrrole radical anion:

6	0.017838000	-1.102737000	-0.313225000
6	0.018858000	-0.710765000	1.014713000
6	0.018863000	0.710765000	1.014713000
6	0.017835000	1.102737000	-0.313225000
7	0.017283000	0.000000000	-1.095283000
1	0.016000000	0.000000000	-2.238943000
1	0.017504000	-2.084494000	-0.780407000
1	0.019583000	-1.363684000	1.883120000

1	0.019592000	1.363684000	1.883120000
1	0.017497000	2.084494000	-0.780407000

Furan radical cation:

6	-0.362015000	-1.081289000	0.000000000
6	0.991356000	-0.684383000	0.000000000
6	0.985001000	0.693478000	0.000000000
6	-0.372004000	1.077869000	0.000000000
8	-1.170159000	-0.005393000	0.000000000
1	-0.842326000	-2.048438000	0.000000000
1	1.838864000	-1.350561000	0.000000000
1	1.826318000	1.367456000	0.000000000
1	-0.861178000	2.040568000	0.000000000

Furan radical anion:

6	-0.369157000	-1.144854000	-0.110460000
6	0.963838000	-0.691630000	0.004549000
6	0.957417000	0.700475000	0.004546000
6	-0.379703000	1.141382000	-0.110454000
8	-1.213918000	-0.005608000	-0.005609000
1	-0.783236000	-2.037720000	0.360464000
1	1.836489000	-1.331485000	0.008528000
1	1.824127000	1.348355000	0.008524000
1	-0.802001000	2.030392000	0.360464000

i1

6	1.207275000	0.688957000	-0.002412000
6	0.001899000	1.381429000	0.011211000
6	-1.218075000	0.703254000	0.015333000
6	-1.195403000	-0.692361000	0.011221000
6	0.006984000	-1.390011000	-0.002402000
6	1.214506000	-0.701195000	-0.010211000
1	2.141214000	1.235949000	-0.003135000
1	0.007853000	2.464737000	0.021433000
1	-2.130597000	-1.239173000	0.021451000
1	0.000246000	-2.472321000	-0.003117000
1	2.151455000	-1.242142000	-0.017830000
6	-2.522802000	1.456541000	-0.002195000
1	-2.431465000	2.423060000	0.494005000
1	-2.852255000	1.646834000	-1.027234000
1	-3.314201000	0.894125000	0.493883000

i1’

6	1.159660000	0.744232000	-0.072797000
6	0.001829000	1.426506000	-0.128946000
6	-1.288211000	0.758338000	-0.020029000
6	-1.286598000	-0.755022000	-0.159508000

6	0.043683000	-1.406671000	0.095655000
6	1.178241000	-0.701137000	0.107228000
1	2.103090000	1.272492000	-0.122700000
1	0.004396000	2.507167000	-0.203103000
1	-2.058097000	-1.194500000	0.476479000
1	0.058940000	-2.482105000	0.222040000
1	2.130830000	-1.193829000	0.254032000
6	-2.418089000	1.450740000	0.177950000
1	-1.585488000	-1.008499000	-1.186875000
1	-2.411924000	2.532030000	0.220054000
1	-3.374967000	0.960377000	0.300883000

i1(-)

6	1.224372000	0.680117000	-0.054458000
6	0.006742000	1.421980000	-0.093220000
6	-1.234100000	0.689231000	-0.006412000
6	-1.210211000	-0.685680000	0.045339000
6	0.003781000	-1.431773000	0.026155000
6	1.233634000	-0.694208000	-0.003038000
1	2.171635000	1.214450000	-0.079230000
1	0.009218000	2.504175000	-0.028310000
1	-2.158769000	-1.218683000	0.091169000
1	-0.004189000	-2.511558000	0.106488000
1	2.182464000	-1.224050000	0.004474000
6	-2.526002000	1.451589000	-0.001642000
1	-2.577210000	2.162651000	0.838703000
1	-2.643798000	2.060241000	-0.913089000
1	-3.388680000	0.783704000	0.070778000

i1(-)’

6	1.219889000	0.717813000	0.047082000
6	0.009246000	1.422833000	0.046168000
6	-1.257277000	0.790593000	0.001763000
6	-1.288092000	-0.740813000	0.098971000
6	0.051223000	-1.410317000	-0.072518000
6	1.220799000	-0.693611000	-0.042256000
1	2.160608000	1.257452000	0.062834000
1	0.027152000	2.509952000	0.044954000
1	-1.735757000	-1.008191000	1.077662000
1	0.070460000	-2.493284000	-0.150714000
1	2.169997000	-1.219366000	-0.111422000
6	-2.462232000	1.452454000	-0.082575000
1	-2.014911000	-1.124363000	-0.634400000
1	-2.511731000	2.535166000	-0.124129000
1	-3.402078000	0.913802000	-0.107345000

i1(+)

6	1.203772000	0.731420000	-0.030349000
6	0.018766000	1.407630000	-0.025540000
6	-1.231928000	0.693569000	0.007357000
6	-1.215209000	-0.735341000	0.036012000
6	-0.028115000	-1.409255000	0.031199000
6	1.194637000	-0.683303000	-0.002026000
1	2.145768000	1.260692000	-0.055424000
1	-0.002533000	2.489162000	-0.046712000
1	-2.155766000	-1.268116000	0.060787000
1	-0.003202000	-2.489819000	0.052173000
1	2.132221000	-1.224165000	-0.005342000
6	-2.497529000	1.444317000	0.014464000
1	-2.533077000	2.102658000	0.893898000
1	-2.533771000	2.133238000	-0.839350000
1	-3.376445000	0.806901000	0.008232000

i1(+)'

6	1.176380000	0.721995000	-0.022278000
6	-0.021471000	1.420455000	-0.016199000
6	-1.266528000	0.751270000	0.006098000
6	-1.273976000	-0.749363000	0.023369000
6	0.047576000	-1.403565000	0.015479000
6	1.212678000	-0.690808000	-0.006308000
1	2.107619000	1.273080000	-0.039725000
1	-0.008009000	2.502570000	-0.028752000
1	-1.840873000	-1.117212000	0.892084000
1	0.070331000	-2.485977000	0.027698000
1	2.166501000	-1.199761000	-0.011498000
6	-2.433635000	1.474403000	0.011363000
1	-1.860183000	-1.136535000	-0.823963000
1	-2.416150000	2.555659000	-0.001306000
1	-3.402964000	0.993910000	0.028011000

i2

6	1.205965000	0.702480000	-0.158886000
6	0.012607000	1.403274000	0.010804000
6	-1.141040000	0.641078000	0.196905000
6	-0.017646000	-1.336916000	0.057339000
6	1.194813000	-0.683641000	-0.135978000
1	2.135413000	1.239434000	-0.307626000
1	-2.095820000	1.139900000	0.333633000
1	-0.063273000	-2.420088000	0.080820000
1	2.106438000	-1.251492000	-0.265110000
7	-1.170042000	-0.691179000	0.221690000
6	-0.035648000	2.906385000	-0.004949000
1	0.326251000	3.304686000	-0.954993000
1	-1.051963000	3.269783000	0.143430000

1	0.589531000	3.331362000	0.782921000

i2'

6	1.267252000	0.699130000	-0.084660000
6	0.049884000	1.465536000	0.125848000
6	-1.155463000	0.651234000	0.550012000
6	-0.100286000	-1.301478000	-0.186318000
6	1.203459000	-0.636043000	-0.211748000
1	2.205372000	1.230874000	-0.191307000
1	-2.080422000	1.137006000	0.237013000
1	-0.137052000	-2.352496000	-0.468802000
1	2.087489000	-1.230008000	-0.402197000
7	-1.202969000	-0.744900000	0.122098000
6	0.013144000	2.794436000	-0.025081000
1	-0.906773000	3.354598000	0.080745000
1	0.907909000	3.358333000	-0.255735000
1	-1.190954000	0.627187000	1.647929000

i2(-)

6	1.237274000	0.732543000	-0.166970000
6	-0.018801000	1.399494000	0.012266000
6	-1.152517000	0.644443000	0.197814000
6	-0.005126000	-1.342376000	0.058394000
6	1.189194000	-0.693288000	-0.134509000
1	2.158385000	1.281332000	-0.316179000
1	-2.103744000	1.161333000	0.333283000
1	-0.023782000	-2.431379000	0.080385000
1	2.094452000	-1.281584000	-0.261689000
7	-1.222039000	-0.727537000	0.232485000
6	-0.082580000	2.899259000	-0.004373000
1	0.285099000	3.310356000	-0.956059000
1	-1.104391000	3.258044000	0.144374000
1	0.548630000	3.340848000	0.780780000

i2(-)'

6	1.259706000	0.708449000	0.075682000
6	0.078338000	1.467759000	-0.092691000
6	-1.152475000	0.648451000	-0.485546000
6	-0.067022000	-1.324038000	0.156604000
6	1.195126000	-0.691514000	0.134448000
1	2.202662000	1.223730000	0.236790000
1	-1.200556000	0.644061000	-1.594665000
1	-0.104254000	-2.389945000	0.397041000
1	2.088596000	-1.283504000	0.300826000
7	-1.227213000	-0.734962000	-0.024765000
6	-0.021551000	2.832562000	0.003580000
1	-0.968118000	3.340281000	-0.137813000

1	0.843288000	3.450912000	0.220216000
1	-2.058240000	1.176421000	-0.164773000

i2(+)

6	1.170461000	0.699388000	0.171310000
6	-0.020301000	1.421872000	0.024741000
6	-1.189161000	0.665456000	-0.209760000
6	0.009688000	-1.388120000	-0.075971000
6	1.209921000	-0.683362000	0.105424000
1	2.091103000	1.242262000	0.347440000
1	-2.150716000	1.101654000	-0.453218000
1	-0.092814000	-2.464663000	-0.016518000
1	2.119151000	-1.247303000	0.263829000
7	-1.037412000	-0.628846000	-0.232270000
6	-0.076790000	2.918518000	0.015677000
1	-1.097917000	3.288995000	-0.027262000
1	0.390782000	3.306486000	0.923125000
1	0.474060000	3.319149000	-0.836546000

i2(+)’

6	1.221912000	0.727858000	0.224506000
6	0.047853000	1.435454000	-0.114210000
6	-1.188467000	0.643325000	-0.390947000
6	-0.028421000	-1.358175000	0.011252000
6	1.187347000	-0.649567000	0.287170000
1	2.137845000	1.266421000	0.432081000
1	-1.587698000	0.888387000	-1.389185000
1	-0.032392000	-2.441724000	0.066451000
1	2.077000000	-1.209628000	0.545098000
7	-1.140727000	-0.776777000	-0.304953000
6	0.057229000	2.815416000	-0.183567000
1	-0.831209000	3.377802000	-0.441317000
1	0.960454000	3.375727000	0.019630000
1	-2.012439000	0.974143000	0.262925000

i3

6	0.000000000	0.692939000	1.215100000
6	0.000000000	1.309996000	-0.041911000
6	0.000000000	-1.318620000	-0.052038000
6	0.000000000	-0.688275000	1.189369000
1	0.000000000	2.391445000	-0.126384000
1	0.000000000	-2.398950000	-0.131452000
1	0.000000000	-1.270859000	2.102073000
7	0.000000000	-0.667513000	-1.209999000
7	0.000000000	0.661016000	-1.199397000
6	0.000000000	1.496232000	2.482766000
1	0.878254000	2.142514000	2.535934000

1	-0.878254000	2.142514000	2.535934000
1	0.000000000	0.851747000	3.360815000

i3’

6	-0.112508000	0.717370000	1.289381000
6	-0.267453000	1.385348000	-0.044999000
6	0.422716000	-1.223301000	-0.018913000
6	0.444782000	-0.616069000	1.176502000
1	0.697834000	1.810342000	-0.353433000
1	0.815067000	-2.215126000	-0.187535000
1	0.824122000	-1.123240000	2.055251000
7	-0.284710000	-0.700185000	-1.137581000
7	-0.658445000	0.484333000	-1.163341000
6	-0.416929000	1.293044000	2.456828000
1	-0.873536000	2.272546000	2.506415000
1	-0.207010000	0.797617000	3.396133000
1	-0.984842000	2.203199000	-0.030050000

i3(-)

6	0.010485000	0.737128000	1.199481000
6	0.163909000	1.311205000	-0.048261000
6	-0.090271000	-1.309803000	-0.049017000
6	-0.128361000	-0.696145000	1.198129000
1	0.274598000	2.396378000	-0.115851000
1	-0.190813000	-2.395461000	-0.110667000
1	-0.256824000	-1.271202000	2.107406000
7	0.059666000	-0.716576000	-1.226651000
7	0.196478000	0.688163000	-1.235331000
6	-0.010791000	1.531967000	2.464280000
1	0.108664000	2.600299000	2.262262000
1	-0.951544000	1.405035000	3.024428000
1	0.791255000	1.235676000	3.159854000

i3(-)’

6	-0.110816000	0.689363000	1.310032000
6	-0.211780000	1.365779000	-0.043419000
6	0.389675000	-1.227790000	-0.073813000
6	0.370882000	-0.638596000	1.188668000
1	-0.871231000	2.237074000	-0.003008000
1	0.768348000	-2.238246000	-0.202274000
1	0.641829000	-1.215751000	2.067237000
7	-0.201194000	-0.711270000	-1.176175000
7	-0.663700000	0.516780000	-1.160825000
6	-0.397358000	1.359676000	2.462383000
1	-0.758402000	2.380816000	2.445035000
1	-0.270720000	0.895340000	3.434903000
1	0.797704000	1.749509000	-0.298409000

i3(+)

6	0.011195000	0.731542000	1.189411000
6	0.173945000	1.375206000	-0.049458000
6	-0.098090000	-1.361160000	-0.030144000
6	-0.124705000	-0.660154000	1.169276000
1	0.290364000	2.439015000	-0.188693000
1	-0.194731000	-2.429674000	-0.142259000
1	-0.253147000	-1.215584000	2.089832000
7	0.063399000	-0.615184000	-1.127975000
7	0.182176000	0.590546000	-1.122581000
6	-0.012416000	1.522349000	2.459794000
1	0.107901000	2.586973000	2.277113000
1	-0.957273000	1.363104000	2.982507000
1	0.787832000	1.189685000	3.123241000

i3(+)’

6	-0.125047000	0.720026000	1.259007000
6	-0.423237000	1.436208000	-0.039922000
6	0.295982000	-1.344428000	0.017737000
6	0.010801000	-0.719012000	1.179671000
1	-1.483297000	1.411337000	-0.312488000
1	0.490672000	-2.388771000	-0.150240000
1	-0.025262000	-1.306956000	2.089216000
7	0.479311000	-0.484902000	-1.084413000
7	0.235497000	0.655881000	-1.149536000
6	0.003381000	1.387989000	2.414539000
1	0.006711000	2.469114000	2.453415000
1	0.091399000	0.862575000	3.356223000
1	-0.073673000	2.463620000	-0.082876000

i4

6	0.010733000	0.000000000	1.304899000
6	-0.010223000	1.174108000	-0.626505000
6	-0.009730000	0.000000000	-1.375879000
6	-0.010223000	-1.174108000	-0.626506000
1	0.023593000	0.000000000	2.388881000
1	-0.024637000	2.141580000	-1.120094000
1	-0.024637000	-2.141580000	-1.120094000
7	0.001349000	1.187723000	0.704782000
7	0.001349000	-1.187723000	0.704781000
6	0.004297000	0.000000000	-2.877621000
1	1.027157000	-0.000042000	-3.261827000
1	-0.494550000	-0.882415000	-3.278451000
1	-0.494478000	0.882457000	-3.278450000

i4’

6	-0.085424000	0.016003000	1.288001000
6	0.102122000	1.290442000	-0.658427000
6	-0.097021000	0.011954000	-1.433886000
6	0.096127000	-1.202116000	-0.644863000
1	-0.231772000	-0.082218000	2.359150000
1	-0.536467000	2.088833000	-1.039530000
1	0.202297000	-2.148058000	-1.175060000
7	-0.109418000	1.177497000	0.780387000
7	0.123320000	-1.225111000	0.634828000
6	-0.414221000	-0.060468000	-2.729009000
1	-0.486072000	-1.016529000	-3.232671000
1	-0.611405000	0.822824000	-3.322941000
1	1.132331000	1.643652000	-0.796791000

i4(-)

6	0.000560000	0.003560000	1.306534000
6	-0.063200000	1.200183000	-0.648618000
6	0.000023000	-0.002359000	-1.364908000
6	0.062411000	-1.181073000	-0.649881000
1	0.001115000	0.000297000	2.400887000
1	-0.114348000	2.163557000	-1.144113000
1	0.112933000	-2.140472000	-1.157660000
7	-0.062727000	1.200278000	0.751765000
7	0.064325000	-1.209858000	0.745740000
6	-0.000041000	-0.008155000	-2.871019000
1	-0.905234000	0.461564000	-3.272966000
1	0.850976000	0.553492000	-3.273373000
1	0.053207000	-1.027156000	-3.261526000

i4(-)’

6	-0.082646000	-0.012281000	1.315552000
6	0.128880000	1.270112000	-0.646709000
6	0.128443000	-0.048169000	-1.414859000
6	0.067935000	-1.210627000	-0.620851000
1	-0.190793000	-0.048317000	2.403044000
1	-0.658939000	1.925799000	-1.052457000
1	0.089265000	-2.179210000	-1.120742000
7	-0.034940000	1.181729000	0.799204000
7	-0.027455000	-1.232307000	0.724324000
6	0.196654000	-0.041674000	-2.788404000
1	0.240283000	0.884735000	-3.348949000
1	0.208385000	-0.964675000	-3.357815000
1	1.067253000	1.810601000	-0.863061000

i4(+)

6	-0.006137000	0.002677000	1.400363000
6	-0.001666000	1.163939000	-0.638435000

6	0.007017000	-0.003002000	-1.420770000
6	0.013931000	-1.165686000	-0.634685000
1	-0.014279000	0.004815000	2.479359000
1	0.001607000	2.154227000	-1.085338000
1	0.030069000	-2.157040000	-1.078026000
7	-0.009386000	1.104662000	0.671559000
7	0.005319000	-1.101454000	0.676617000
6	-0.000746000	-0.000908000	-2.910871000
1	-0.994138000	0.255172000	-3.286082000
1	0.701412000	0.734245000	-3.304169000
1	0.266999000	-0.977788000	-3.308663000

i4(+)’

6	-0.250408000	0.010052000	1.320428000
6	0.287661000	1.305076000	-0.650468000
6	0.171708000	0.015187000	-1.450437000
6	0.285421000	-1.178018000	-0.621962000
1	-0.617553000	-0.081629000	2.333576000
1	-0.310712000	2.100566000	-1.103084000
1	0.445948000	-2.144716000	-1.098467000
7	-0.153171000	1.090138000	0.694801000
7	0.177396000	-1.167165000	0.649643000
6	-0.022270000	-0.066485000	-2.772104000
1	-0.251896000	0.804487000	-3.372661000
1	0.048634000	-1.013718000	-3.291646000
1	1.321565000	1.661942000	-0.609342000

i5

6	1.170579000	0.636517000	0.028505000
6	-0.030655000	1.310732000	-0.158899000
6	-1.179102000	-0.648869000	-0.011811000
6	0.035832000	-1.314652000	0.175654000
1	2.115198000	1.167544000	0.042988000
1	-0.049599000	2.386355000	-0.295291000
1	0.057382000	-2.390351000	0.312211000
7	-1.199200000	0.676962000	-0.179621000
7	1.205749000	-0.682952000	0.196778000
6	-2.491370000	-1.375104000	-0.033215000
1	-2.999021000	-1.210775000	-0.984702000
1	-2.359890000	-2.446312000	0.111964000
1	-3.147445000	-0.993806000	0.750498000

i5’

6	1.136283000	0.603653000	0.210724000
6	-0.075289000	1.298086000	-0.264846000
6	-1.266306000	-0.628397000	0.035058000
6	0.018770000	-1.405065000	-0.077747000

1	2.004711000	1.203693000	0.476809000
1	-0.005223000	2.349534000	-0.533987000
1	0.185483000	-1.700779000	-1.122564000
7	-1.220760000	0.731776000	-0.322210000
7	1.212322000	-0.658498000	0.332143000
6	-2.428371000	-1.148810000	0.437049000
1	-2.497550000	-2.170121000	0.784274000
1	-3.326229000	-0.547466000	0.425506000
1	-0.029895000	-2.330845000	0.494418000

i5(-)

6	1.162499000	0.645203000	0.042576000
6	-0.018396000	1.299153000	-0.193964000
6	-1.157704000	-0.666769000	-0.010791000
6	0.031695000	-1.312152000	0.225288000
1	2.092750000	1.211830000	0.057821000
1	-0.008130000	2.375137000	-0.363355000
1	0.025043000	-2.388237000	0.394984000
7	-1.249026000	0.688863000	-0.235889000
7	1.264804000	-0.703100000	0.267526000
6	-2.461059000	-1.415659000	-0.038533000
1	-2.960581000	-1.295191000	-1.007605000
1	-2.318577000	-2.483037000	0.147661000
1	-3.153858000	-1.020304000	0.714279000

i5(-)’

6	1.156621000	0.559873000	-0.207098000
6	-0.026265000	1.313937000	-0.049719000
6	-1.274244000	-0.598869000	0.166611000
6	0.024399000	-1.292740000	0.564140000
1	2.059826000	1.074952000	-0.542399000
1	0.018044000	2.398011000	-0.138666000
1	-0.038038000	-2.365103000	0.355948000
7	-1.246881000	0.762813000	0.055176000
7	1.233668000	-0.744864000	-0.043990000
6	-2.415248000	-1.320888000	-0.014756000
1	-2.413481000	-2.400093000	0.067298000
1	-3.349577000	-0.824948000	-0.247169000
1	0.121180000	-1.203090000	1.665029000

i5(+)

6	1.201715000	0.655324000	0.040850000
6	-0.031787000	1.332928000	-0.153068000
6	-1.201453000	-0.672517000	-0.014993000
6	0.061355000	-1.341795000	0.180575000
1	2.140204000	1.196150000	0.059625000
1	-0.058383000	2.408767000	-0.286643000

1	0.083226000	-2.417910000	0.313859000
7	-1.120340000	0.621014000	-0.164979000
7	1.147621000	-0.637751000	0.193209000
6	-2.495964000	-1.421442000	-0.040514000
1	-2.992494000	-1.256632000	-0.997681000
1	-2.334649000	-2.485785000	0.103347000
1	-3.149594000	-1.044612000	0.747299000

i5(+)’

6	1.151183000	0.528751000	-0.332794000
6	-0.093094000	1.341049000	-0.076553000
6	-1.253304000	-0.631856000	0.230290000
6	0.027496000	-1.297231000	0.689364000
1	2.012741000	0.980858000	-0.814917000
1	-0.028256000	2.420698000	0.022961000
1	0.015841000	-2.346043000	0.379924000
7	-1.181539000	0.722720000	0.103528000
7	1.176759000	-0.642485000	0.114633000
6	-2.399107000	-1.295728000	-0.081197000
1	-2.432980000	-2.375952000	-0.099230000
1	-3.308434000	-0.746103000	-0.281975000
1	0.162700000	-1.299686000	1.776371000

i6

6	0.000000000	-1.120189000	-0.325614000
6	0.000000000	-0.708194000	0.984448000
6	0.000000000	0.717979000	0.996588000
6	0.000000000	1.120663000	-0.318566000
7	0.000000000	-0.000296000	-1.112032000
1	0.000000000	0.002103000	-2.115054000
1	0.000000000	-2.106747000	-0.755271000
1	0.000000000	-1.355922000	1.845802000
1	0.000000000	2.108598000	-0.746405000
6	0.000000000	1.605821000	2.201782000
1	-0.878762000	1.434700000	2.828207000
1	0.878761000	1.434699000	2.828208000
1	0.000001000	2.658308000	1.916221000

i6’

6	-0.141528000	-1.088903000	-0.306159000
6	-0.297633000	-0.694163000	0.971909000
6	-0.113005000	0.746715000	1.044553000
6	0.310491000	1.171812000	-0.357550000
7	0.080387000	-0.032399000	-1.177902000
1	0.710524000	-0.191435000	-1.947955000
1	-0.217792000	-2.090964000	-0.702102000
1	-0.573665000	-1.327726000	1.798479000

1	1.368844000	1.450812000	-0.371708000
6	-0.288127000	1.563563000	2.089288000
1	-0.594229000	1.180887000	3.053177000
1	-0.131059000	2.630940000	2.011355000
1	-0.267583000	2.017078000	-0.731132000

i6(-)

6	0.016041000	-1.106562000	-0.321406000
6	0.034550000	-0.718684000	1.008009000
6	0.053438000	0.705035000	1.019511000
6	0.045201000	1.096690000	-0.309265000
7	0.022687000	-0.002466000	-1.097524000
1	0.012050000	0.018130000	-2.220147000
1	-0.001769000	-2.088349000	-0.786734000
1	0.035081000	-1.378228000	1.873531000
1	0.053495000	2.082127000	-0.773166000
6	0.077469000	1.608921000	2.217666000
1	-0.796744000	1.470016000	2.864250000
1	0.960950000	1.447368000	2.846136000
1	0.087820000	2.657525000	1.907454000

i6(-)’

6	-0.049561000	-1.172141000	-0.350866000
6	-0.257565000	-0.701700000	0.958160000
6	-0.154221000	0.698295000	1.043143000
6	0.208386000	1.155800000	-0.361615000
7	-0.060052000	-0.008453000	-1.233662000
1	0.656385000	-0.087136000	-1.942452000
1	-0.498424000	-2.083872000	-0.743790000
1	-0.495281000	-1.343696000	1.798058000
1	1.280243000	1.442758000	-0.395134000
6	-0.253700000	1.584535000	2.107454000
1	-0.485212000	1.244910000	3.110337000
1	-0.183436000	2.654646000	1.953271000
1	-0.361555000	2.039582000	-0.680213000

i6(+)

6	-0.108826000	-1.099070000	-0.328654000
6	-0.183920000	-0.694574000	1.006902000
6	-0.008088000	0.677816000	1.047525000
6	0.178828000	1.095825000	-0.332291000
7	0.112205000	0.014066000	-1.115819000
1	0.207676000	0.008089000	-2.121151000
1	-0.195301000	-2.080854000	-0.766528000
1	-0.350195000	-1.351513000	1.843883000
1	0.346356000	2.085663000	-0.728756000
6	0.000476000	1.592844000	2.208544000

1	-0.158521000	1.056670000	3.140070000
1	0.951530000	2.130454000	2.269869000
1	-0.776665000	2.356106000	2.104718000

i6(+)'

6	-0.149458000	-1.084728000	-0.333127000
6	-0.368258000	-0.647944000	0.980393000
6	-0.134289000	0.740760000	1.028627000
6	0.257114000	1.179316000	-0.362839000
7	0.204003000	-0.074995000	-1.107080000
1	0.411639000	-0.153622000	-2.092613000
1	-0.238297000	-2.087919000	-0.723837000
1	-0.664755000	-1.279394000	1.801868000
1	1.262709000	1.604819000	-0.409330000
6	-0.236590000	1.555016000	2.123567000
1	-0.523902000	1.152088000	3.084485000
1	-0.035988000	2.615785000	2.066262000
1	-0.437922000	1.904346000	-0.793687000

i7

6	-0.339665000	-1.092209000	0.000112000
6	0.960982000	-0.715278000	0.000342000
6	0.973753000	0.722282000	-0.000056000
6	-0.332231000	1.090818000	-0.000344000
8	-1.149425000	-0.001404000	-0.000340000
1	-0.832903000	-2.047592000	0.000216000
1	1.815748000	-1.370867000	0.000697000
1	-0.823803000	2.047441000	-0.000664000
6	2.172625000	1.614894000	-0.000004000
1	2.796863000	1.442446000	0.879327000
1	2.797510000	1.441683000	-0.878722000
1	1.882435000	2.665353000	-0.000563000

i7'

6	-0.314209000	-1.064207000	-0.143519000
6	0.948652000	-0.681155000	-0.372628000
6	1.035291000	0.751311000	-0.139438000
6	-0.372934000	1.151269000	0.263342000
8	-1.156782000	-0.070164000	0.232388000
1	-0.766346000	-2.041563000	-0.209941000
1	1.758614000	-1.322320000	-0.676690000
1	-0.428842000	1.557081000	1.273806000
6	2.090186000	1.564262000	-0.243315000
1	3.059500000	1.187580000	-0.538510000
1	2.016098000	2.623553000	-0.038018000
1	-0.830487000	1.857739000	-0.429797000

i7(-)

6	-0.341822000	-1.152767000	0.111760000
6	0.957703000	-0.683214000	-0.138825000
6	0.978165000	0.699910000	0.036403000
6	-0.326560000	1.104863000	0.409102000
8	-1.179395000	-0.031069000	0.293343000
1	-0.834025000	-2.012786000	-0.336451000
1	1.815541000	-1.309281000	-0.351112000
1	-0.805422000	2.034475000	0.091080000
6	2.169922000	1.598968000	-0.017144000
1	2.432473000	2.015365000	0.969500000
1	3.049167000	1.067556000	-0.392583000
1	2.006143000	2.465547000	-0.675073000

i7(-)’

6	-0.341047000	-1.155525000	0.010954000
6	0.954892000	-0.701046000	-0.286776000
6	1.045107000	0.694692000	-0.159331000
6	-0.348676000	1.121451000	0.265305000
8	-1.194719000	-0.029557000	0.162364000
1	-0.846139000	-2.010130000	-0.433604000
1	1.778014000	-1.353717000	-0.546734000
1	-0.352815000	1.476229000	1.314246000
6	2.090846000	1.598285000	-0.283798000
1	3.092725000	1.279124000	-0.544900000
1	1.927008000	2.662949000	-0.168186000
1	-0.766455000	1.930633000	-0.351861000

i7(+)

6	-0.351385000	-1.074658000	-0.066931000
6	0.979436000	-0.697766000	-0.163628000
6	1.024239000	0.682422000	0.005739000
6	-0.340456000	1.066315000	0.202187000
8	-1.147952000	0.009128000	0.156288000
1	-0.855571000	-2.026155000	-0.132395000
1	1.807225000	-1.365402000	-0.337622000
1	-0.801350000	2.028146000	0.373601000
6	2.181374000	1.601824000	-0.003707000
1	2.248149000	2.143069000	0.944814000
1	3.113293000	1.069640000	-0.171936000
1	2.060872000	2.361003000	-0.782308000

i7(+)’

6	-0.343351000	-1.055396000	-0.135220000
6	0.966620000	-0.645910000	-0.370652000
6	1.013056000	0.737886000	-0.136029000
6	-0.372661000	1.150573000	0.263113000

8	-1.134852000	-0.088275000	0.222613000
1	-0.783311000	-2.041098000	-0.206658000
1	1.778537000	-1.286436000	-0.675366000
1	-0.452698000	1.542547000	1.278365000
6	2.108291000	1.563843000	-0.247179000
1	3.069146000	1.166718000	-0.543725000
1	2.043685000	2.624052000	-0.043822000
1	-0.853719000	1.844883000	-0.427762000
